# The water depth-dependent co-occurrence patterns of marine bacteria in shallow and dynamic Southern Coast, Korea

**DOI:** 10.1038/s41598-019-45512-5

**Published:** 2019-06-24

**Authors:** Yingshun Cui, Seong-Jun Chun, Seung Ho Baek, Minji Lee, Yunji Kim, Hyung-Gwan Lee, So-Ra Ko, Seungwoo Hwang, Chi-Yong Ahn, Hee-Mock Oh

**Affiliations:** 1 Cell Factory Research Center, Korea Research Institute of Bioscience and Biotechnology (KRIBB), Daejeon, Republic of Korea; 2 Department of Environmental Biotechnology, KRIBB School of Biotechnology, Korea University of Science and Technology (UST), Daejeon, Republic of Korea; 3 South Sea Institute, Korea Institute of Ocean Science & Technology (KIOST), Geoje, Republic of Korea; 4 Korean Bioinformation Center (KOBIC), KRIBB, Daejeon, Republic of Korea

**Keywords:** Microbial ecology, Environmental microbiology

## Abstract

To investigate the interactions between bacterial species in relation to the biotic and abiotic environmental fluctuations, free-living (FL), nanoparticle-associated (NP), and microparticle-associated (MP) bacterial community compositions (BCCs) were analyzed. A total of 267 samples were collected from July to December 2016 in the dynamic and shallow southern coastal water of Korea. The variations in BCC mostly depended on planktonic size fraction. Network analysis revealed water depth-dependent co-occurrence patterns of coastal bacterial communities. Higher interspecies connectivity was observed within FL bacteria than NP/MP bacteria, suggesting that FL bacteria with a streamlined genome may need other bacterial metabolites for survival, while the NP/MP copiotrophs may have the self-supporting capacity to produce the vital nutrients. The analysis of topological roles of individual OTUs in the network revealed that several groups of metabolically versatile bacteria (the marine Roseobacters, *Flavobacteriales*, *Desulfobacterales*, and SAR406 clade) acted as module hubs in different water depth. In conclusion, interspecies interactions dominated in FL bacteria, compared to NP and MP bacteria; modular structures of bacterial communities and keystone species strongly depended on the water depth-derived environmental factors. Furthermore, the multifunctional, versatile FL bacteria could play pivotal roles in dynamic shallow coastal ecosystems.

## Introduction

Planktonic bacteria often dominate the biomass in oceans and make large contributions to biogeochemical cycles. Based on the bacterial relationship with particulate matter in oceans, planktonic microbes have been widely classified into the free-living (FL) and the particle-associated (PA) bacteria. FL bacteria tend to have a relatively smaller genome size^1^ and contribute more to carbon fixation, N and P uptake and iron acquisition^2^. Particulate organic matters (POMs), mainly organic aggregates and fecal pellets, are hot spots for PA bacteria^3,4^. Metabolic activities of PA bacteria vary widely, including chemolithoautotrophic CO_2_ fixation, seawater methane production and oxidation, denitrification, anaerobic ammonium oxidation, sulfate reduction, sulfur oxidation, etc.^5^. Many studies have focused on the identification of FL and PA bacterial community compositions (BCCs) and found that FL and PA bacteria are composed of significantly different members^6–8^. FL bacteria are generally dominated by highly abundant taxa with streamlined genomes, such as SAR11 and SAR86, while PA bacteria dominated by *Bacteroidetes*, *Planktomycetes*, *Verrucomicrobia*, etc. In addition, some bacterial groups can switch their lifestyles depending on chemical triggers and substrate availability^9^. Although some consistent taxa have been detected in FL and PA, it remains difficult to compare the studies due to the lack of consensus on the filter pore size used to separate FL and PA bacteria^10^.

The genetic diversity and activity of planktonic marine bacteria are significantly influenced by the surrounding environmental conditions. Many studies have shown that BCC depends on physical conditions (e.g., temperature and salinity) and resource availability (e.g., nutrients)^11–15^. Ecological interactions within microbiomes are other factors that could influence BCC by competing for resources, exchanging genetic materials and facilitating metabolic cooperation^5,16,17^. As an emerging approach, correlation-based microbial network analysis could decipher the potential interconnectivities of microbes in relation to environmental fluctuations^18–22^. These studies have documented microbial interaction mechanisms, but far less focus has been placed on the size fraction-based microbial interactions^11^. In addition, the repeatability of the observed correlations within microbiomes has not been explored fully.

A coastal ecosystem could provide diverse environments that would be suitable for exploring the interspecies connectivities within bacterial assemblages, both FL and PA bacteria, in relation to natural and anthropogenic perturbations. The shallow Tongyeong-Geoje coastal area is occasionally affected by water mass exchange in the open western Pacific Ocean and the East China Sea. Many fish and oyster farms are located in this area (up to 263,525 m^2^ in Tongyeong coast in 2013, according to Statistics Korea)^23^, discharging waste into the seawater^24^. Harmful algal blooms, especially of *Cochlodinium polykrikoides*, have frequently occurred in this area over the last two decades from July to August. Sampling was conducted in this dynamic and shallow coastal area from June to December 2016, the presumed period of pre-bloom/bloom/post-bloom. The main objectives of this study were 1) to identify the spatiotemporal patterns of FL and PA BCC, 2) to explore the microbial interconnectivity and the topological features of microbial networks, and 3) to seek the keystone microbes that provide the greatest contributions to the stabilization of microbial communities and functions.

## Results

### Environmental parameters and phytoplankton community compositions

The water depth of study area ranged from 30 m to 65 m, depending on the sampling locations. Seawater samples were collected from three different depths (surface, middle, and bottom layers) at six different sites (Supplementary Fig. S1). The middle-water was collected at a half depth of each site and the bottom-water at a depth of 1 m above from the sediment. Seawater temperature, salinity, nutrient concentrations, and Chlorophyll-*a* (Chl-*a*) concentrations are summarized in Supplementary Fig. S2. Temperature and salinity ranged from 14.1 °C to 29.7 °C and 29.0 psu to 34.4 psu, respectively. Generally, the surface temperature (average: 21.8 ± 4.5 °C) was higher than the bottom layer temperature (average: 18.2 ± 3.4 °C), and the surface salinity (average: 32.1 ± 1.4 psu) was lower than the bottom layer salinity (average: 33.4 ± 1.0 psu). Higher surface temperature and lower salinity were usually recorded in August (up to 29.7 °C and as low as 29.0 psu). Relatively stable temperature and salinity were observed in September and December. Nitrite and nitrate nitrogen (NOx), phosphate, and silicate concentrations were generally higher in the bottom than the surface layer, while dissolved oxygen (DO) and pH showed the opposite patterns. NOx concentrations in the bottom layer ranged from 0.17 μM to 12.04 μM, generally higher than those in the surface (ranged from 0.02 μM to 6.77 μM). Station NT26, the shallowest station in this study, showed relatively low variations of physicochemical factors in different sampling depth compared to other stations. The phytoplankton cell density, as well as their community composition, varied considerably from month to month and from surface to bottom layer (Supplementary Fig. S3). The surface and middle layers typically contained a higher phytoplankton cell density compared to the bottom layer. *Pseudo-nitzschia* and *Chaetoceros* blooms, which made up more than 80% of the total phytoplankton, were observed at a middle-depth of station NT20 in July (6.8 × 10^5^ cells L^−1^) (Supplementary Fig. S3b) and from surface water of NT20 in October (8.5 × 10^5^ cells L^−1^) (Supplementary Fig. S3a), respectively.

### Bacterial variations in diversity, richness, and evenness

The alpha-diversity (measured by Shannon’s diversity index) in the nanoparticle-associated (NP) (4.51 ± 0.54) and microparticle-associated (MP) (4.45 ± 0.64) fractions was higher than in the FL fraction (4.00 ± 0.45) from the total fractions (Supplementary Fig. S4a) (one-way ANOVA and Tukey test: *P* < 0.05). The highest species richness (Chao1 richness estimator) was observed in the NP fraction (324.97 ± 136.47) from the total size fractions (Supplementary Fig. S4e), while the larger size fractions showed significantly higher evenness (Simpson’s evenness index) (Supplementary Fig. S4i) (one-way ANOVA and Tukey test: *P* < 0.05). The identical patterns were maintained from each size fraction (Supplementary Fig. S4b–d, f–h, and j–l). These three indices also showed water depth dependencies: the bottom layer showed a higher species richness and alpha-diversity in all size fractions. The species evenness seemed to be less affected by the water depth: only the MP fraction of the bottom layer showed significantly higher evenness compared with other two fractions. Importantly, alpha-diversity and richness indices were positively correlated with the nutrient concentrations (e.g., NOx, phosphate, and silicate) but negatively with DO (%) and pH (Supplementary Table S1).

### Bacterial community structure depending on size fraction

After removing low-quality sequences, chimeras, and singletons/doubletons/tripletons/contaminants, a total of 5,891,450 sequences were obtained from 267 samples. NMDS 2-dimensional plots, based on a metric of dissimilarity among the samples, revealed that BCC varied mostly depending on the size fraction (perMANOVA test: pseudo *F* = 40.90, *P* < 0.001) (Supplementary Table S2): samples from the same size fraction clustered together, but FL bacterial samples clustered more closely (Supplementary Fig. S5). Within the same size fraction, BCC showed a pattern indicative of temporal succession across the samples (Supplementary Fig. S6). BCC showed relatively little variation in September and December, while it varied considerably in July and August, when thermocline was observed in the water column. In addition, samples from the bottom layer tended to maintain similar BCCs, especially in the NP and MP fractions, but larger variations were observed in the surface samples.

Approximately 80% of the obtained sequences were affiliated with *Alphaproteobacteria*, *Gammaproteobacteria*, *Deltaproteobacteria*, *Flavobacteriia*, and *Sphingobacteriia* (Supplementary Fig. S7). The average relative abundance of *Alphaproteobacteria*, SAR406, and *Acidimicrobiia* in FL samples (43 ± 11%, 5 ± 3%, and 4 ± 2%, respectively) decreased approximately 2 to 5 times in NP and MP fractions. However, *Deltaproteobacteria* and *Planctomycetacia* increased approximately 4 to 5 times with the larger size fractions (MP fraction: 9 ± 5% and 5 ± 4%, respectively). *Gammaproteobacteria* and *Flavobacteriia* dominated in all size fractions. BCC also varied largely from month to month. For instance, significantly higher proportions of *Sphingobacteriia* and *Verrucomicrobiae* were detected in PA fractions only in August (up to 45% and 25%, respectively). Higher proportions of *Cyanobacteria* were detected in PA fractions in summer and early autumn (up to 22%), while *Planctomycetacia* was more enriched in PA fractions in September (ca. 5–10%).

**Figure 1:**
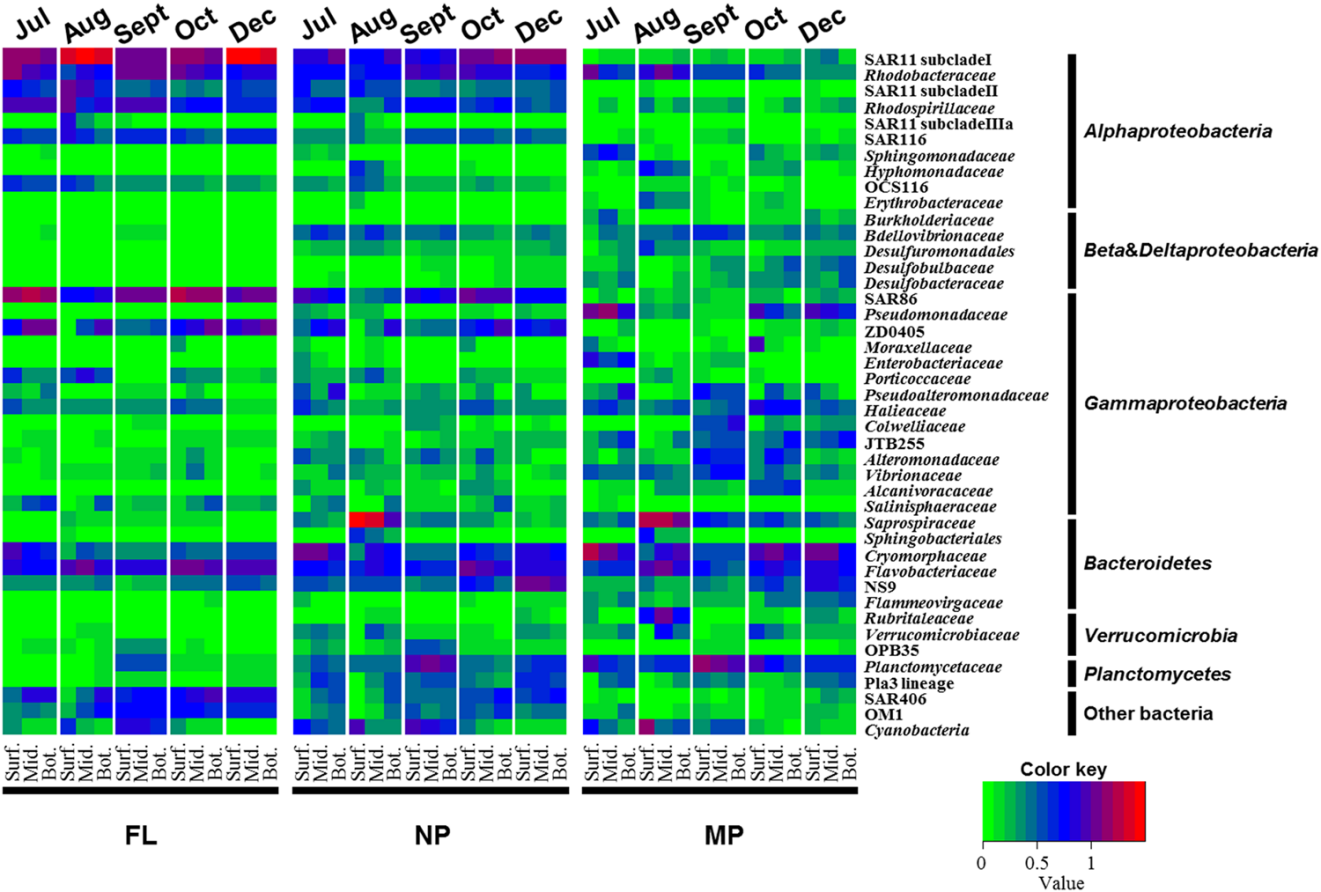
A heatmap of the 10 most enriched families in each sample. Average values of families within each fraction from different water depths are listed. Surf, surface; Mid, middle depth; Bot, bottom depth.

At the family level, SAR11 subclade I/subclade II/subclade IIIa, *Rhodospirillaceae*, and SAR116 were more enriched in the FL fraction (22 ± 13%, 6 ± 3%, 3 ± 1%, respectively) and decreased with the larger size fractions: < 1% on average in the MP fraction (Fig. 1). SAR86 (11 ± 5%), ZD0405 (*Oceanospirillales*, 6 ± 4%), SAR406 (5 ± 3%), and OM1 (*Acidimicrobiia*, 3 ± 2%) also showed similar patterns. More diverse taxa with lower variance were observed in the larger size fractions compared with the FL fraction. *Cryomorphaceae*, *Planctomycetaceae*, and *Verrucomycetaceae*, together with many families assigned to *Gammaproteobacteria* and *Deltaproteobacteria* (e.g., *Bdellovibrionaceae*, *Halieaceae*, *Saprospiraceae, Pseudomonadaceae*, etc.), were more enriched in PA fractions, especially in the MP fraction (3–7% on average). *Rhodobacteraceae* and *Flavobacteriaceae* dominated in all fractions. BCC also varied depending on the water depth. SAR11 subclade II/subclade IIIa, SAR 116, *Halieaceae* in the FL fraction and *Cyanobacteria* and *Planctomycetaceae* in the PA fractions were found principally in the surface layer, while ZD0405 (*Oceanospirillales*), SAR406, and *Salinisphaeraceae* in the FL fraction and JTB255 (*Xanthomonadales*), *Desulfobulbaceae*, and *Desulfobacteraceae* in NP or MP fractions were more enriched in the bottom layer (one-way ANOVA followed by Tukey test: *P* < 0.05).

### Potential drivers of BCC

**Figure 2:**
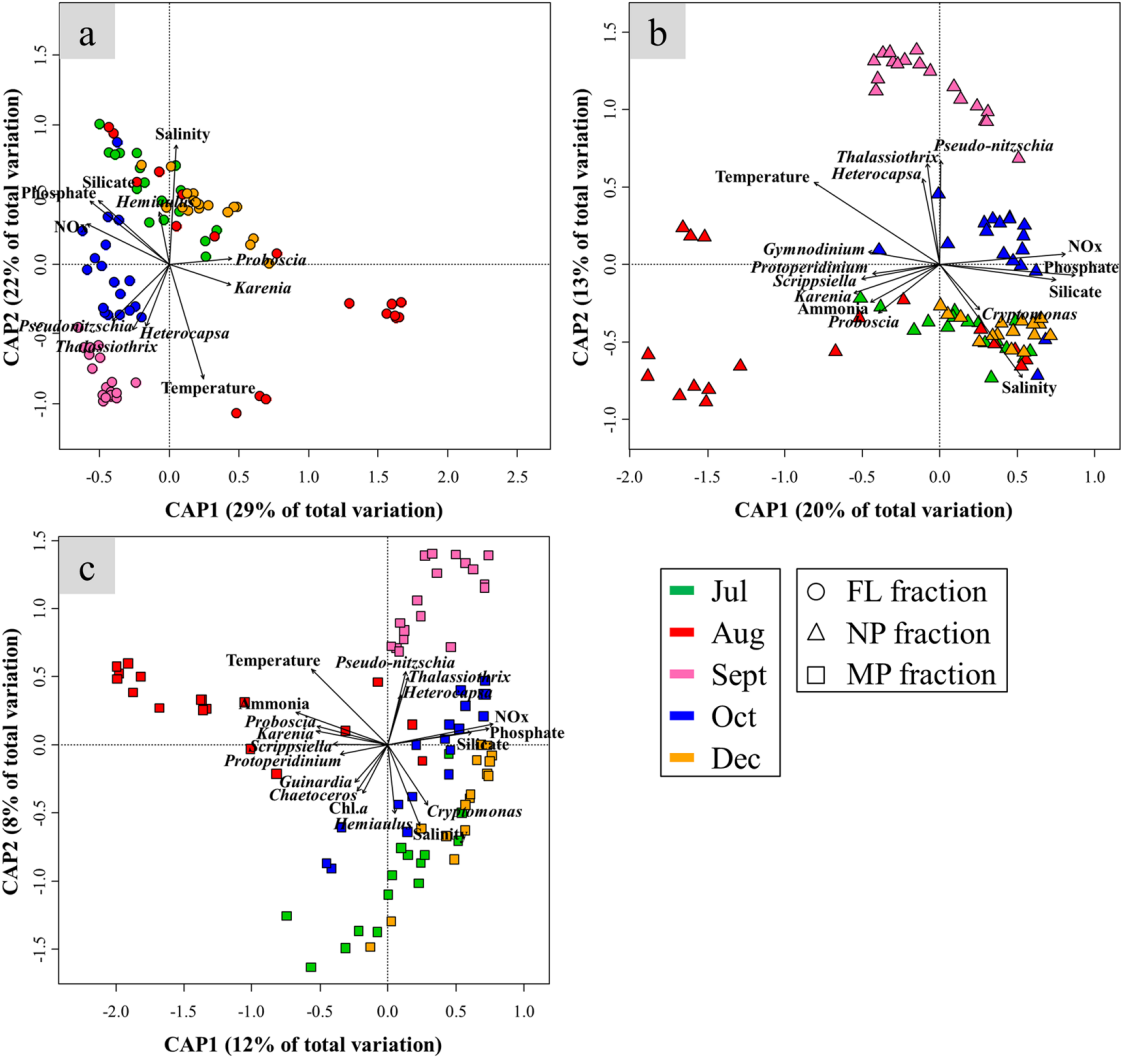
Distance-based redundancy analysis (db-RDA) in the FL (**a**), NP (**b**), and MP (**c**) fractions. All three db-RDAs and environmental parameters included were statistically significant (*P* < 0.001).

**Figure 3:**
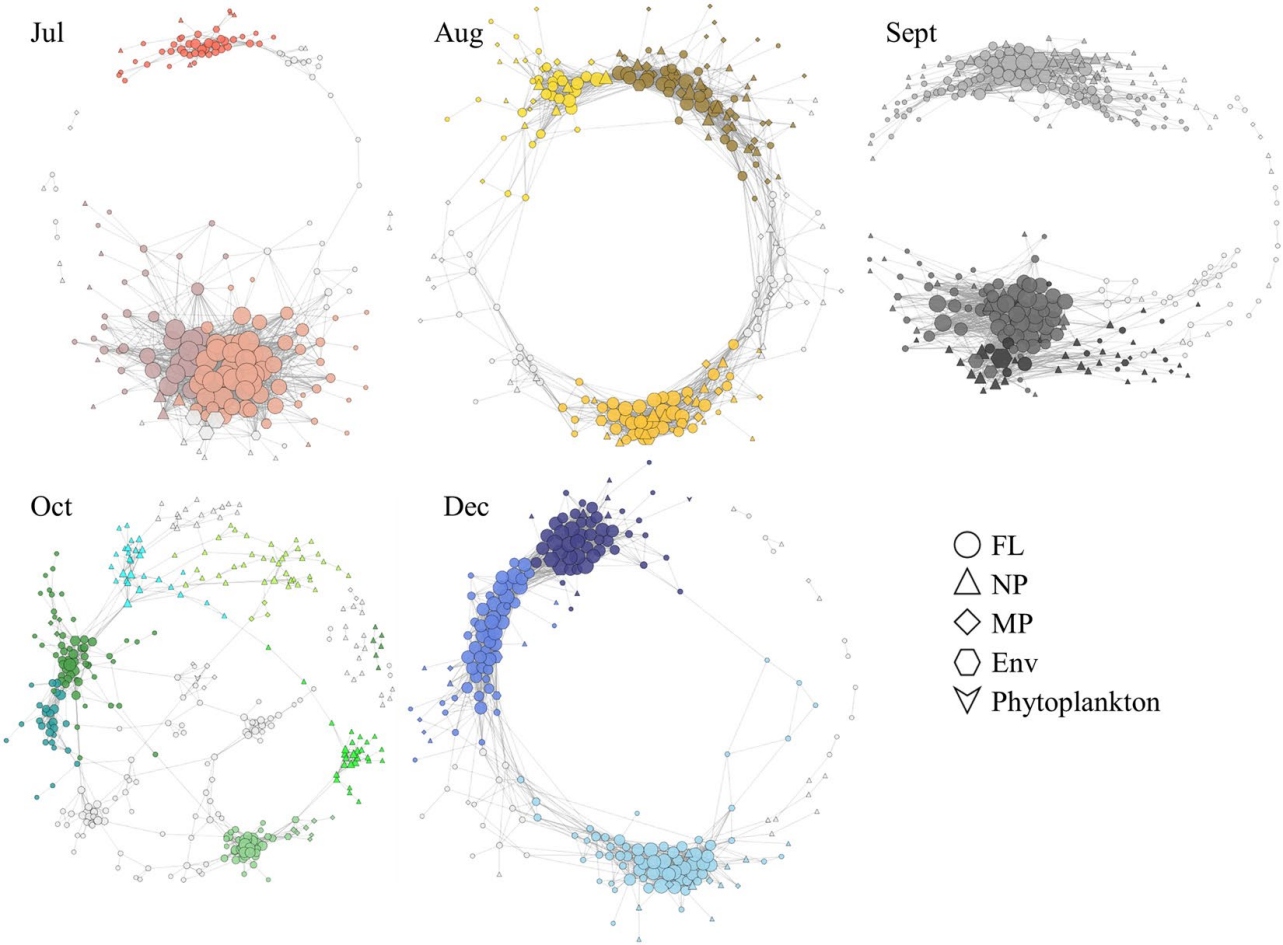
Correlation-based co-occurrence networks for each month. The size of each node is proportional to the number of connections (i.e., degree). Different colors indicate different modules in each month.

Distance-based redundancy analysis (db-RDA) was performed to evaluate the effects of biotic and abiotic environmental parameters on bacterial community structure in each size fraction (Fig. 2). The db-RDA results showed that salinity and temperature were the most influential parameters in all size fractions. The relative contributions of NOx, phosphate, and silicate concentrations increased in the NP and MP fractions. The explainable proportions in db-RDA decreased with increasing size fraction (FL, 51%; NP, 33%; MP, 20%), which indicated that other factors that were not measured could be more important in reshaping BCC in larger size fractions. The fluctuation of the phytoplankton community seemed to have a less effect on BCC compared with the abiotic environmental parameters. A serious phytoplankton bloom did not occur in the Tongyeong-Geoje coastal area in 2016. Comparatively higher concentrations of *Pseudo*-*nitzschia* and *Chaetoceros* were observed only at one site (NT20), in July and October, respectively (Supplementary Fig. S3). However, BCC patterns were not so different within the same size fraction from all sampling sites, even including site NT20, in each month (Supplementary Fig. S7). Furthermore, BCC-phytoplankton correlations were seldom found in co-occurrence networks (Fig. 3). Even though the db-RDA visually showed that there could be significant relationships between phytoplankton and BCC (Fig. 2), the correlation-based network analysis statistically showed that phytoplankton and BCC were not so closely related, compared with bacteria-bacteria and bacteria-environment (Fig. 3). It could be summarized that phytoplankton community changes could be much less influential to BCC than the abiotic environmental conditions, at least when a large phytoplankton bloom did not occur.

### Co-occurrence networks and their topological and taxonomic features

The monthly network was constructed 1) to explore bacterial co-occurrence patterns both inter-/intra-connectivities among different size fractions and 2) to evaluate the individual bacterial susceptibility to the environmental factors (Fig. 3). After filtering (*Q* < 0.01 and *ρ* ≥ 0.7 but *Q* < 0.05 and *ρ* ≥ 0.7 for October), a total of 8,554 out of 218,199 (approximately 4%) correlations were statistically significant among 1,303 variables (the combined values of monthly networks). In general, the number of significant correlations within FL bacteria (6.5 ± 2.8%) and between FL bacteria and environmental factors (8.1 ± 3.4%) outnumbered the sum of correlations of those in other size fractions (Supplementary Table S3). These results reflected a higher number of co-occurrence instances in the FL bacteria (edge/node ratio = 10 ± 3) compared to NP (edge/node ratio = 5 ± 3) and MP (edge/node ratio = 3 ± 3) and indicated the higher susceptibility of FL bacteria to the ambient environmental conditions.

**Figure 4:**
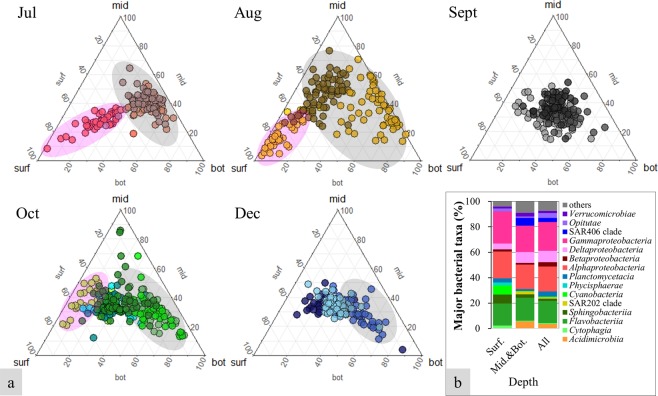
Ternary plots of bacterial OTUs in different modules with reference on the water depth (**a**) and bacterial composition (class level) of major modules in different water depths (**b**). Different colors indicate different modules. Modules composed of the OTUs dominating in surface were shaded with pink ovals, middle and bottom depth with gray ovals. Surf., surface depth; Mid.&Bot., middle and bottom depth; All, all depths.

The topological features of monthly co-occurrence networks are summarized in Table 1. For all months, the coastal microbial networks showed higher values of modularity (0.24–0.78), clustering coefficient (CC) (0.52–0.73) and average path length (APL) (2.93–6.58) than those in their respective Erdös–Rényi random networks, indicating that coastal microbial networks had small-world properties (that is, microbes are more interconnected than a random network with similar size) and modular structures. Therefore, if highly connected hub microbes were lost in “a small world”, it could cause catastrophic damage to the whole network. Some modules were composed of OTUs relatively evenly distributed in the whole water depths (defined as W-modules), but some of the modules reflected the water depth-dependent patterns, i.e., tending to be more abundant in the specific water depth (surface: S-modules; middle and bottom water layers: MB-modules) (Figs. 3 and 4). OTUs assigned to *Cyanobacteria*, *Phycisphaerae*, *Planctomycetacia*, *Cytophagia*, *Sphingobacteriia*, and *Opitutae* preferred to co-occur in S-modules, while OTUs assigned to *Acidimicrobiia*, *Deltaproteobacteria*, SAR202 clade, SAR406 clade, and *Verrucomicrobia* preferred to co-occur in MB-modules. More MB-modules were observed in the half-year compared to S-modules, mainly due to the typhoon-induced mixing of the water column from summer to autumn, which affected the surface water more profoundly compared to the bottom water despite the shallowness of the sampling area (30 m to 65 m).

**Table 1:** Comparison of topological properties of monthly association networks of bacterial communities with identically sized Erdös–Rényi random networks.

Topological properties	Jul	Aug	Sept	Oct	Dec
Nodes	186	233	301	355	228
Edges	1858	1779	1945	1096	1876
Diameter	14	10	8	17	12
Network density	0.11	0.07	0.04	0.02	0.07
Average node degree	19.98	15.27	12.92	6.17	16.46
Modularity	0.24	0.61	0.51	0.78	0.60
Modularity, random	0.18	0.20	0.24	0.34	0.20
Clustering coefficient (CC)	0.73	0.60	0.52	0.54	0.63
Clustering coefficient, random (CCr)	0.11	0.07	0.04	0.02	0.07
Ratio of CC/CCr	6.84	9.14	11.68	33.58	8.60
Average shortest path length (APL)	5.58	3.98	2.93	6.58	4.02
Average shortest path length, random (APLr)	1.99	2.28	2.51	3.44	2.21
Ratio of APL/APLr	2.80	1.75	1.17	1.91	1.82
Small-word coefficient σ, (CC/CCr)/(APL/APLr)	2.45	5.23	10.00	17.55	4.73

*σ > 1 indicates small-world network properties, meaning the bacteria were highly and efficiently connected with each other (Telesford *et al*.^67^).

The node degree distribution of the co-occurrence networks showed a scale-free power law distribution (*R*^2^ = 0.35–0.84). Consequently, the top 10% most highly connected OTUs occupied 68 ± 14% of total observed correlations, even though most of them occupied a low relative abundance among the total bacteria (ca. 0.05% to 1.5% on average). A wide variety of taxonomic groups were observed, mainly including *Flavobacteriales* (17%), *Oceanospirillales* (10%), and SAR406 clade (9%). Co-occurrence analysis revealed that OTUs from the same phyla tended to co-occur more frequently (ranging from 6.0 ± 2.6% to 43.8 ± 35.8%) than from different phyla, except *Proteobacteria* (Supplementary Table S4). Notably, all phyla but *Bacteroidetes* showed high inter-phylum correlations with SAR406 clade. The proportions of *Proteobacteria*-*Proteobacteria* (6.0 ± 2.6%) were relatively lower than the inter-phylum correlations with *Actinobacteria* (8.1 ± 4.4%), *Planctomycetes* (7.1 ± 7.8%), and SAR406 clade (10.9 ± 7.2%), despite being abundant and ubiquitous in coastal water. More correlations were observed between environmental factors and SAR406 clade, *Actinobacteria*, and *Planctomycetes*, indicating a high sensitivity of these bacteria to the fluctuations of the surrounding environment.

**Figure 5:**
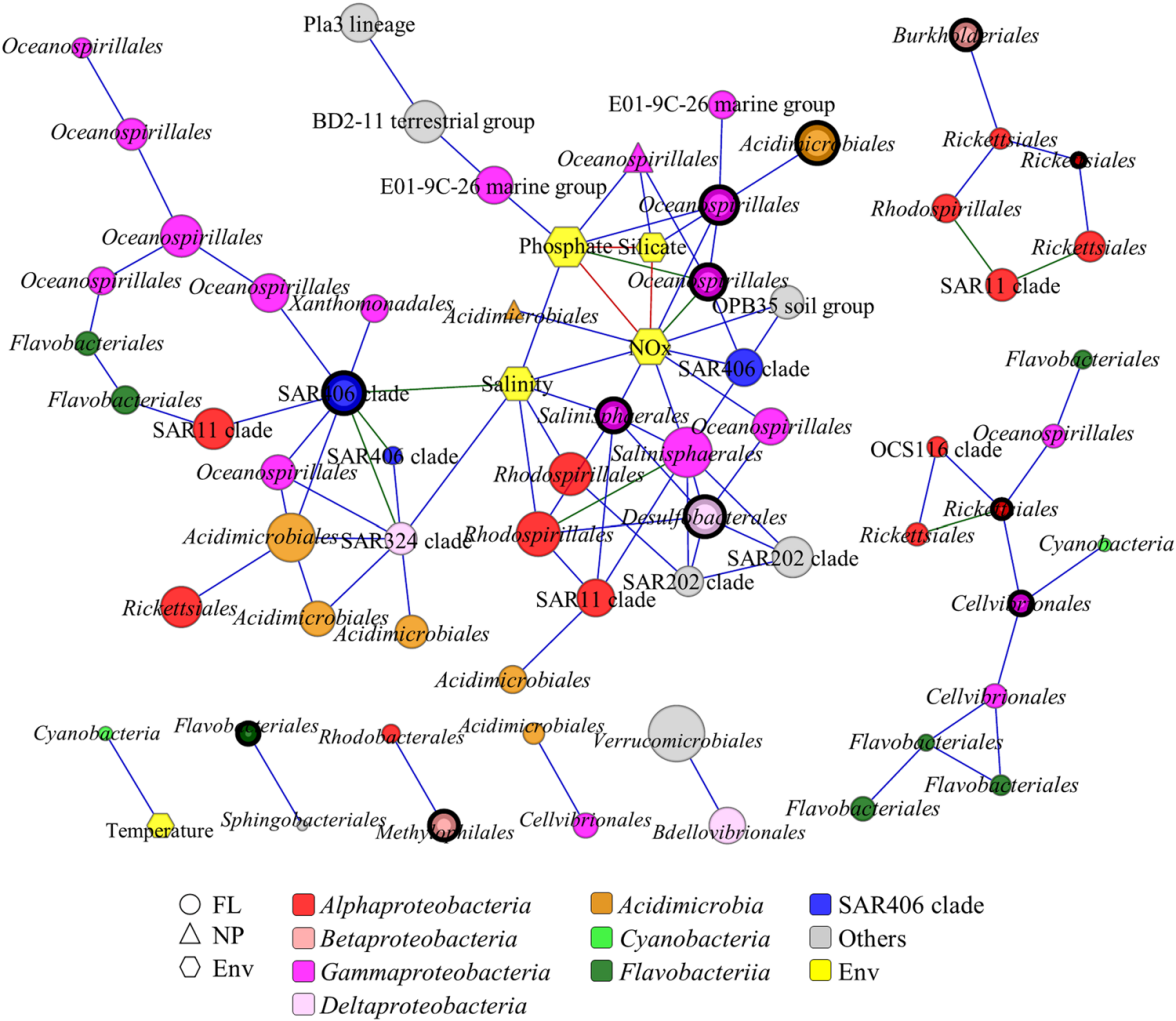
The recurrence-based network constructed with correlations observed more than 3 times in monthly networks (edge color: 3 times, blue lines; 4 times, green lines; 5 times, red lines). The order name of an OTU is denoted in the node. The size of each node is proportional to the average degree over five months (monthly networks). OTUs are colored by phylum level. Module hubs are marked with thick black outlines.

The correlations that appeared more than 3 times between two identical variables in monthly co-occurrence networks were collected to construct the recurrence-based co-occurrence network (Fig. 5). Consequently, 95 correlations (approximately 1% in total correlations) among 65 OTUs and 4 abiotic environmental parameters (temperature, NOx, phosphate, silicate) met the criteria. OTUs assigned to *Oceanospirillales*, *Flavobacteriales*, and *Rickettsiales* co-occurred repeatedly with OTUs in the same order. OTUs assigned to SAR406 clade, *Oceanospirillales*, *Rhodospirillales*, and *Salinisphaerales* showed repeated correlations with environmental factors, including salinity, phosphate, silicate, and NOx concentrations. One OTU assigned to *Cyanobacteria* (*Synechococcus*) also showed a repeatable correlation with temperature. No repeatable correlation was observed in OTUs of MP fractions, most like due to the uniqueness of BCC in each particles (to be discussed later).

### Network roles of OTUs and their taxonomical properties

**Figure 6:**
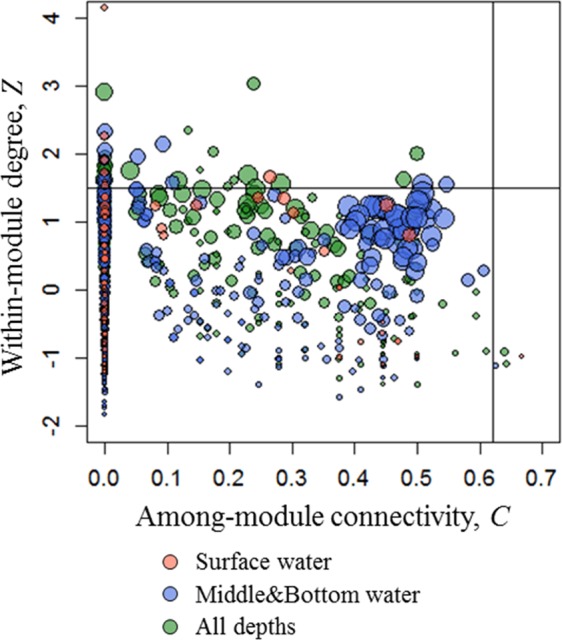
Plot of within-module degree (*Z*) and among-module connectivity (*C*). The threshold values of *Z* and *C* for categorizing OTUs were 1.5 and 0.62. The different colors indicate that OTUs were more abundant at specific water depths: red, surface depth; blue, middle and bottom depth; green, whole depths. The symbol size is proportional to the node degree.

In an ecological network, roles of individual nodes reflect the potential importance of OTUs in the microbial community. Within-module degree (*Z*) and among-module connectivity (*C*) can describe the relatedness of a node with other nodes in the same module and with other nodes in other modules, respectively. A higher *Z* score of a node indicates an essential role in its own module compared to other nodes. If a node has all its links within its own module, then *C* = 0; if the links of a node are distributed evenly among modules, then *C → *1. Therefore, the roles of a node could be defined by its position in the *ZC*-parameter space (Fig. 6). A total of 52 module hubs (approximately 4% in total OTUs) were observed (Supplementary Table S5). Since over 80% of module hubs were either uncultivated or unclassified at genus level, we analyzed the module hubs at order level in this study. The taxonomical positions of most module hubs (e.g., *Flavobacteriales* and *Rhodobacterales*) seemed to be less affected by the water depth-related parameters. However, *Desulfobacterales*, SAR406 clade, *Salinisphaerales*, and OM190 clade were only observed as module hubs in MB-modules. Connections within module hubs and between module hubs and non-module-hub OTUs showed repeatable co-occurrence patterns in the half-year (Fig. 5). To name a few, OTUs assigned to SAR406 clade co-occurred with OTUs assigned to *Oceanospirillales*, SAR11 clade, or *Xanthomonadales*; two module-hub OTUs assigned to *Salinisphaerales* and *Desulfobacterales* co-occurred repeatedly. In addition, module hubs (OTUs assigned to SAR406 clade, *Oceanospirillales*, and *Salinisphaerales*) strongly correlated with environmental factors, such as salinity, phosphate, silicate, and NOx concentrations.

### Metagenome-functional predictions

Microbial putative functions based on BCC were analyzed by the functional annotation of prokaryotic taxa (FAPROTAX) to evaluate the potential functional differences between different size fractions. An average 37 ± 9% of obtained sequences was assigned to the functional groups of FAPROTAX database. Aerobic chemoheterotrophy was the most abundant function in all size fractions (responsible for an average 34% to 37% of total functions in different size fractions). The putative functions of BCC varied monthly in different size fractions (perMANOVA tests: pseudo *F* = 14.51, *P* < 0.001) (Supplementary Fig. S8). More abundant putative functions were observed in larger size fractions compared to the FL fraction. The functions of degradation of aromatic compounds and transformation of sulfur and nitrogen were both much more abundant in the NP and MP fractions compared to the FL fraction. Bacteria belonging to *Pseudomonadales* and *Oceanospirillales* were inferred to contribute to the former function and bacteria belonging to *Desulfobacterales*, *Planctomycetales*, *Xanthomonadales*, and *Burkholderiales* to the latter function. Methanol oxidation, which could be attributed to *Methylococcales*, was more abundant in the FL fraction.

## Discussion

Bacterial diversity varies depending on the planktonic size fraction. Many studies have focused on uncovering whether FL or PA bacteria contain higher diversity and species richness, but contradictory results have been observed, even in the same marine area: Acinas *et al*.^25^ and Ghiglione *et al*.^26^ reported higher diversity in the FL fraction, while Crespo *et al*.^27^ observed increasing bacterial diversity in larger size fractions. A recent study that separated six discrete size fractions revealed an increased bacterial diversity in larger size fractions^10^. In our study, the diversity indices, as well as species richness, were higher in the PA fraction, particularly in the NP fraction (Supplementary Fig. S4). The species evenness increased with larger size fractions. BCCs in the MP fraction could differ markedly depending on the particle features. The origins of particles vary from fish feces and various detritus to small zooplankton and phytoplankton. The individual particle is most likely to be dominated by specific bacteria adapted to their own particular environment. Considering the huge numbers as well as the uniqueness of each particle, the diversity indices of the bacterial community from NP/MP fractions would exceed those from FL fractions. The diversity and species richness increased with nutrient concentrations (NOx, phosphate, and silicate) but decreased with pH and DO (Supplementary Table S1). The nutrient concentrations were higher in the bottom layer, while pH and DO were higher on the surface (Supplementary Fig. S2). Therefore, a greater number of bacterial species were adapted to more eutrophic conditions. It can be concluded that bacterial diversity and species richness are influenced significantly by bacterial lifestyles. Water depth-derived physicochemical parameters also seem to play essential roles in determining these indices.

SAR86, an uncultivated lineage of *Gammaproteobacteria*^28^, was also detected at an average of 6% in the NP fraction. Similar results have been reported previously^13,29^, indicating either environmental adaptations to eutrophic conditions (because particles usually contain more available nutrients) or the presence of eutrophic ecotypes. SAR11, especially SAR11 subclade I, was enriched in the surface FL fraction (Fig. 1). The genomic studies revealed that SAR11 contains a streamlined, small genome size and is genetically adapted to the FL lifestyle^28,30,31^. The relatively higher proportion of SAR11 subclade I and subclade II in the NP fraction could be explained by two possibilities. First, the filtration system itself could result in the artefact, because large particles might clog the filters, making obligate free-living SAR11 remain in the PA fractions, even though the filters were replaced regularly during filtration. Second, there could be distinct PA ecotypes not yet cultivated, which might occupy a niche in association with bacterioplankton or other planktonic organisms, as reported by other size fraction studies^10,32^. In addition, particle-associated SAR11 proportions increased in December when the sampling area was highly influenced by the Kuroshio Current, which introduces warm and high-salinity water from the open ocean, implying environment-specific adaptations of these SAR11 ecotypes. The proportions of SAR11 subclade I, subclade II and subclade IIIa in the FL fraction increased in August when relatively low-salinity and low-nutrient but high-temperature water was introduced, most likely originating from a large discharge of the Changjiang due to heavy rainfall in summer^33^. SAR11 subclade IIIa, which is known to be more abundant in oligohaline–mesohaline environments^34,35^, occupied up to 9% of the total bacteria, suggesting an ecotype-specific sensitivity among SAR11 groups against salinity and temperature.

*Bdellovibrionaceae*, *Cryomorphaceae*, and *Saprospiraceae* were more enriched in NP and MP fractions. These bacterial groups are known to degrade marine algae-derived polysaccharides or directly assimilate particulate organic nitrogen and proteins^36,37^. The particles generally contain much higher concentrations of C/N/P in comparison to the seawater, thereby supplying a eutrophic environment for the adaptation of these bacteria to the PA lifestyle. Some bacteria assigned to *Bdellovibrionaceae* (e.g., *Bdellovibrio*) were reported to be obligate predators that prey on other environmental microbes. Due to the higher abundance of microbial cells per volume in PA fractions than in FL fractions, they could make predation on other microbes more efficiently. This could be another reason for the observation in higher abundance of predators in NP and MP fractions in this study. FAPROTAX-based functional prediction also revealed that the microbial predatory or exoparasitic processes were more enriched on marine particles (Supplementary Fig. S8). It further supports the prey-predator hypothesis. The potential anaerobic sulfate reducing bacteria or metal oxide reducing bacteria (e.g., *Desulfobulbaceae* and *Desulfobacteraceae*) were more enriched in the NP and MP fractions. The functional prediction showed that most anaerobic metabolic processes (e.g., sulfate respiration and fermentation) were found on marine particles. Marine particles could harbour hypoxic and even anoxic microenvironments^38^, thus facilitating anaerobic metabolic processes in the oxic bulk seawater.

Previous correlation-based association networks have focused more on the overall trend of microbial interconnectivity. Such an approach might have overlooked temporal interspecies interactions in response to environmental fluctuations. We constructed both monthly and recurrence-based co-occurrence networks 1) to explore the co-occurrence patterns of bacteria in a dynamic coastal water, 2) to identify and compare potential keystone species (the highly correlated OTUs, including the module hubs and connectors) and their taxonomic positions and 3) to evaluate the repeatability of the correlations within taxa in the half year. Recently, Needham *et al*.^39^ reported that oligotypes could provide more detailed profiles of microbial community compositions and thus be more useful for identifying interconnectivities within microbes, including bacteria and viruses. According to their report, when a distinct OTU dominated in a specific time, a single amplicon sequence variant (ASV) accounted for a major proportion in most cases. It suggests that the network constructed with traditional OTUs could not result in intrinsically different outcomes compared to the network constructed with ASV. Consistent with the previous finding^6^, interspecies interaction dominated in FL bacteria, less so in NP and MP bacteria (Fig. 3). Far less bacteria-phytoplankton interaction was observed compared to bacteria-bacteria interactions.

PA bacteria have larger genome sizes on average and more genes related to vitamin synthesis, polysaccharide degradation, and competition^1,2,40^. These genomic characteristics endow PA bacteria with greater adaptability to the copiotrophic lifestyle^41^, allowing them to survive independent of other bacteria. The opposite findings were observed in cyanobacteria-infested fresh water^42,43^, where they demonstrated that bacteria in particles showed higher interconnectivities and also more tightly coupled with each other compared to FL bacteria. This discrepancy might be caused by the different physiological properties of bloom-forming phytoplankton between freshwater and marine water systems. More frequent *Microcystis*-related blooms in freshwater systems could have made bloom-associated bacterial communities relatively stable through long-term adaptation to the ambient microenvironment. Therefore, the large proportions of bloom-associated bacteria and the relative uniformity of PA fractions could form higher interconnectivities in freshwater ecosystem. In contrast, the particles captured in filters could be composed of a wider variety of organic/inorganic materials as well as phytoplankton cells in this study. These different characteristics of freshwater and marine phytoplankton might have brought opposite interaction patterns in this study.

FL bacteria, e.g., SAR11 and SAR86, which are more adapted to oligotrophic conditions, have streamlined genomes compared with copiotrophs^44^. 16S rRNA gene sequence-based functional prediction also showed that the fractions of NP and MP had more diverse functional capabilities than the FL fraction (Supplementary Fig. S8). Although the functional assignment of the coastal ecosystems based on microbial taxonomy is putative, these predictions provide insights into the potential functions of different size fractions. The potentially limited functions due to streamlined genomes made FL bacteria more actively interact with other bacteria to redeem their genetic “weakness” for survival. Evolutionarily, many of the streamlined-genome microbes gained the advantage of faster reproduction by discarding some essential genes, which could be compensated for by the surrounding bacteria (Black Queen Hypothesis)^45^. This survival strategy also made them (the beneficiaries) inevitably rely on neighbouring microbes (the helpers) to supply their needs. Such potential beneficiary (streamlined genome)-helper (multifunctional genome) interaction mechanisms could be one of the reasons why the FL bacteria showed stronger interspecies interactions in the association networks.

Similar to other aquatic ecosystems^19,46,47^, the coastal microbial community in the monthly co-occurrence networks showed a power-law degree distribution, i.e., small-world properties and modular structures (Table 1). Recently, Chafee *et al*.^48^ reported that the correlations within microbes appear relatively stable in a temperate coastal marine environment. In our study, the modular structures of bacterial communities differed between oligotrophic and eutrophic conditions and coupled with certain abiotic or biotic environmental factors (e.g., silicate concentrations, temperature, Chl-*a* concentrations). Therefore, a microbial modular perspective might be more helpful than a specific bacterial species perspective when exploring highly dynamic ecological network. Module formation reflected water depth-dependent patterns (Fig. 4). MB-modules were observed in almost all months, while S-modules were observed clearly only in July and August. No distinguishable water depth-dependent modules were formed in September, when the whole water were mixed due to a typhoon. These results indicated that more conserved bacterial communities with tighter interdependencies could be formed in a more stable environment, even though the water depth of the sampling area was quite shallow (30 m to 65 m). More phototrophs and potential rhizobia (e.g., *Planctomycetes*, *Rhizobiales*, and *Cyanobacteria*) formed modules in the surface water, but more bacteria transferred organic matter, including sulfur/nitrogen compounds (e.g., *Desulfobacterales*, *Desulfuromonadales*, SAR406 clade), in the middle to bottom water layers. Higher nutrient concentrations were observed toward bottom water layers. These nutrients could be good sources for the copiotrophs mentioned above and could be degraded as easy-to-use materials for other bacteria. The surface water characterized by higher temperature and radiation could be a better place for phototrophs and nitrogen-fixing bacteria.

In an ecological network, module hubs function as the fundamental and essential species whose presence could provide minimal growth factors shared among bacterial communities. Module hubs observed in this study were composed of a wide variety of taxonomic groups and reflected water depth dependencies. In the recurrence-based network, module hubs, as well as the high-degree OTUs, showed strong and repeatable interdependencies even though some major environmental changes occurred, such as typhoon-induced mixing of whole water column (from September to October) and seasonal variations of environmental factors (e.g., temperature and salinity). No repeatable correlations were found in the OTUs of MP fraction and only two OTUs of NP fraction showed repeatable correlations with other variables. As we discussed above, the origins of particles could vary significantly, which could also result in the variations in BCC. Since the individual particles could be dominated by specific bacteria that were adapted to their own microenvironment, it was hard to find repeatable correlations among OTUs of NP and MP fractions. Uncultured SAR406 bacteria and the *Desulfobacterales* lineage were only observed as module hubs in the bottom water layers. SAR406 clade is widespread in sub-euphotic zones of the oceans and displays depth-dependent changes^49,50^. The genomic property of *Nitrospina*, one of module hubs in October, has the capacity for oxidization of nitrite to nitrate^51^. These two groups were inferred to transform sulfur/nitrogen compounds and complex, high-molecular-weight organic matters^52^. Higher nutrient concentrations were observed in the bottom water during the sampling periods. Therefore, these potential degraders might be adapted to the more eutrophic conditions (the bottom water layer) and contribute to transforming the organic matters to support other bacteria. In contrast, *Rhodobacterales* (mostly the marine Roseobacters) and the *Flavobacteriales* lineage were observed as module hubs throughout the water depths. Isolates of the marine Roseobacters and *Flavobacteriales* have a relatively large genome size, ~4.5 Mb and ~6.1 Mb, respectively (NCBI genome database). The marine Roseobacters also contain a higher gene content than other marine bacteria^53^, such as SAR11 or SAR86 clades. Further, the marine Roseobacter lineage is responsible for diverse biogeochemical processes, including carbon, nitrogen, phosphorus, and sulfur transformations, several of which are expected to be important for interactions with phytoplankton^54^. The members of *Flavobacteriales* have highly efficient, multiprotein extracellular systems^6^, which could facilitate the use of high-molecular-weight macromolecules present in dissolved organic matters^54^. For instance, *Owenweeksia*, one of module hubs in July and December, has the genes encoding arylsulfatase A family protein^55^, indicating that it could play a role in the degradation of sulfolipids, thus contributing to sulfur cycling in the seawater. Therefore, one of the necessary qualifications for a module hub could be genetically versatile functions, which would be the foundation for involvement in multiple biogeochemical processes in ecological networks and supporting other bacteria.

In conclusion, the variations of BCC mostly depended on planktonic size fraction. *Alphaproteobacteria*, SAR406, and *Actinobacteria* were more adapted to the FL lifestyle, while *Gammaproteobacteria*, *Bacteroidetes*, and *Planctomycetes* were better adapted to the PA lifestyle. Co-occurrence patterns of bacterial communities and module hubs (or keystone species) showed water depth dependency, even though the study area was characterized by shallow depths and dynamic changes to environmental conditions. Higher interspecies connectivity was observed within FL bacteria, indicating that the bacteria with streamlined genomes might need various kinds of essential help from other bacteria to survive. Notably, most of the keystone species have the genetic potential for transforming high-molecular-weight organic matters, which could be used by the streamlined-genome bacteria for growth. Our major conclusions are (1) FL bacteria reflected higher interspecies connectivities, (2) bacterial interaction communities and corresponding keystone species depended on water depth-derived environmental factors, and (3) the multifunctional versatile FL bacteria could play pivotal roles in the dynamic shallow coastal ecosystem.

## Methods

### Sampling sites and sample collection

The sampling was carried out monthly from July to December 2016 (except for November) in the southern coastal area of Korea (detailed description in Supplementary Methods) on board an R/V Jangmok I or a fishing boat. Seawater from the bottom layer was collected approximately 1 m above the seafloor. The middle-layer sample was collected at half depth of each sampling site. The surface-water samples were collected with a clean bucket, while samples from the middle and bottom layers were collected with a 5-L PVC Niskin sampler (General Oceanics, Miami, FL, USA). Once collected, approximately 1–2 L of water samples were immediately filtered through 20-µm-pore-size Nuclepore^®^ filters (Whatman^®^, Clifton, NJ, USA), then through 3.0-µm-pore-size Nuclepore^®^ filters (Whatman^®^, Clifton, NJ, USA), and finally through 0.22-µm-pore-size Sterivex^TM^ filter units (Millipore^TM^, Bedford, MA, USA) to collect MP (>20 µm), NP (3 to 20 µm), and FL (0.22 to 3 µm) bacteria, respectively. The filtration time was no longer than 30 minutes. We replaced the filters when the filtration speed slowed down. The filters were stored at −80 °C until DNA extraction. A total of 267 samples were collected over half a year.

### Environmental parameters

Temperature, salinity, pH, and DO were measured on board using a YSI 6600 data sonde (YSI, USA). Nutrient analysis, Chl-*a* measurements, and enumeration of phytoplankton populations were performed in the laboratory after the cruise (detailed description in Supplementary Methods).

### DNA extraction, sequencing, and sequence processing

Genomic DNA was extracted using the ChargeSwitch^®^ Forensic DNA Purification Kit (Invitrogen, Carlsbad, CA, USA) according to the manufacturer’s instructions^56^. The extracted DNA samples were PCR-amplified for 30 cycles (the first-stage PCR) using the primers below (underlined) appended with Illumina adapter overhang nucleotide sequences (double underlined): 341 F (5′-TCGTCGGCAGCGTCAGATGTGTATAAGAGACAGCCTACGGGNGGCWGCAG-3′) and 805 R (5′-GTCTCGTGGGCTCGGAGATGTGTATAAGAGACAGGACTACHVGGGTATCTAATCC-3′). This primer set targets the V3-V4 region of the bacterial 16S rRNA^57,58^. After purification of the first-stage PCR products using the Ampure system (Agencourt Bioscience Corporation), the second-stage PCR (8 cycles) was performed to attach dual indices and Illumina sequencing adapters to each sample. After purification, the concentrations of PCR products were quantified using the Quant-iT^TM^ PicoGreen^®^ dsDNA Kit (Invitrogen, Carlsbad, CA, USA), pooled in equimolar concentrations and sequenced by high-throughput paired-end Illumina sequencing (MiSeq, 2 × 250 bp reads) at Macrogen Incorporation (Seoul, South Korea). Bacterial 16S rRNA gene sequences and accompanying metadata have been deposited in the Sequence Read Archive (SRA) of NCBI under project number PRJNA431508, PRJNA431510, and PRJNA431050.

The paired-end 16S rRNA gene sequences for taxonomic analysis were processed using mothur v. 1.39.3^59^ following the standard operating procedure (SOP) proposed by Kozich *et al*.^60^. Briefly, low-quality sequences were removed from the analysis if they contained ambiguous characters, more than two mismatches to the forward primer or one mismatch to the barcode, or were under 300 bp or over 500 bp. After removing tripletons, the pre-cluster method^61^ was applied to further reduce the sequencing errors produced by the MiSeq Illumina sequencing platform. Chimeras were identified and removed using chimera.uchime^62^. The average read length was approximately 400 bp after barcode and primer sequences were trimmed. The Silva database (release 123) was used to align and classify the sequences. The sequences were clustered into OTUs at distance threshold of 0.03 using the average neighbour method.

### Statistical analysis

Alpha-diversity, species richness, and evenness were estimated using the Shannon diversity index, Chao1 richness estimators, and Simpson’s evenness index, respectively. All diversity indices were calculated with mothur v. 1.39.3^59^. The differences in indices among FL, NP, and MP bacteria were analyzed by one-way ANOVA followed by Tukey’s test. NMDS and perMANOVA were performed using the Vegan package^63^ in R to compare and evaluate discrepancies between bacterial communities in the FL, NP, and MP fractions and among sampling months, depths, and stations. A heatmap was constructed with the 10 most enriched families in each sample to compare the bacterial community variations in different size fractions in different depths at family level. The average values of families in each fraction from different water layers were used to construct heatmap. The relative abundance of each family was arcsine-transformed before use. db-RDA^64^ was performed with Vegan^63^ to investigate the potential environmental drivers of BCCs in FL, NP, and MP samples. To evaluate the influence of phytoplankton community fluctuations on bacterial community structures, the phytoplankton community data (genus level) were log_10_-transformed before use.

### Network analysis

The co-occurrence network was constructed with the obtained variables, including OTUs, phytoplankton community compositions (genus level), and environmental parameters. OTUs with a maximum relative abundance higher than 0.1% per sample and observed in more than 70% of samples in the same size fraction was selected for analysis. The phytoplankton genera observed in more than 70% of samples were further analyzed. Spearman’s rank correlation coefficients (*ρ*) were calculated in R. The false discovery rates (*Q*-values) were calculated from the observed *P*-value distribution. Only the correlations with *Q*-value < 0.01 (corresponding to *P*-value < 0.002) and *ρ* ≥ 0.7 were retained for construction of the association network. For the October samples, the above criteria obtained 433 correlations among 233 variables, which made up approximately 20% of those obtained in other months. Therefore, the selection criteria was lowered as a *Q*-value < 0.05 (corresponding to *P*-value < 0.001) and *ρ* ≥ 0.7 for October. With these criteria, we obtained approximately 1,100 correlations among 355 variables, which were comparable to other months. Erdös-Rényi random networks, which had the same numbers of nodes and edges as the real co-occurrence networks, were generated with each edge having the same probability of being assigned to any node^65^, and the average value of each network metric was reported. Topological properties of both random and real networks, including CC, APL, average node degree, network density, diameter, and modularity analysis (modules were identified by using the Louvain algorithm), were conducted with the R package igraph^66^. The small-world coefficient (σ) was calculated to investigate the small-world property of the networks (i.e., the degree of clustering and shortness of paths between nodes)^67^. To explore the repeatability of the correlations obtained in monthly association networks, we performed recurrence-based co-occurrence network analysis with the correlations observed more than three times over five months. All networks were visualized with Cytoscape v3.4.0.^68^.

The potential role of individual OTUs (or species) was examined using *Z* and *C* scores^69^. Based on *Z* and *C* scores, each node could be classified into four topological roles: module hubs (*Z* > 1.5 and *C* ≤ 0.62, indicating high connectivity of an OTU in its own module), network hubs (*Z* > 1.5 and *C* > 0.62, indicating high connectivity of an OTU in entire network), connectors (*Z* ≤ 1.5 and *C* > 0.62, a connector OTU that linked different modules, therefore being important to network coherence), and peripherals (*Z* ≤ 1.5 and *C* ≤ 0.62, OTUs that connected with a few other OTUs within/among modules).

### Metagenome-functional predictions and statistical analysis

FAPROTAX^70^ was used to infer the potential functional annotation of taxa in different size fractions. FAPROTAX predicts functions of uncultured prokaryotes from the known functions of cultured bacterial genera. Since the functions represented in FAPROTAX focus on marine and aquatic biogeochemistry, this approach is suitable for interpretation of our data set. However, there are two major limitations in applying this approach: 1) the inferred presumption of FAPROTAX is that if the cultured members of a taxon can perform a particular function, then all members of the same taxon, both cultured and uncultured, can perform that function; 2) the FAPROTAX database is not exhaustive; therefore, only small proportions of OTUs might be assigned to at least one functional group. Despite these caveats, the predicting putative functional groups using FAPROTAX was seen as a useful alternative when metagenomic or metatranscriptomic data were not available.

A heatmap was constructed with putative functional profiles to compare the functional variations in different size fractions in different depths. The monthly averaged value of putative functional profiles in each size fraction in different water depths was calculated. After calculating Euclidean distance among these values, average clustering algorithm was used to visualize the results in a heatmap. The calculation and visualization were performed by using the ‘heatmap.2’ function in gplot. The significance of the observed differences (size fraction, sampling depth, and sampling month) was determined by perMANOVA as described above.

## Supplementary information

The water depth-dependent co-occurrence patterns of marine bacteria in shallow and dynamic Southern Coast, Korea
