# Supplementary material for: The water depth-dependent co-occurrence patterns of marine bacteria in shallow and dynamic Southern Coast, Korea
Source: Sci Rep. 2019 Jun 24;9:9176. doi: 10.1038/s41598-019-45512-5 (PMC6591218; doi:10.1038/s41598-019-45512-5)
Supplement: Supplementary file 1 — The water depth-dependent co-occurrence patterns of marine bacteria in shallow and dynamic Southern Coast, Korea [file 41598_2019_45512_MOESM1_ESM.docx]

# The water depth-dependent co-occurrence patterns of marine bacteria in shallow and dynamic Southern Coast, Korea

**Yingshun Cui, Seong-Jun Chun, Seung Ho Baek, Minji Lee, Yunji Kim, Hyung-Gwan Lee, So-Ra Ko, Seungwoo Hwang, Chi-Yong Ahn & Hee-Mock Oh**

### Supplementary Methods

## Description of the sampling sites

The study sites in the Tongyeong-Geoje coastal area are part of the southern coastal area of Korea (Supplementary Fig. S1). This area is mainly influenced by the Tsushima Current originating from the Kuroshio Current. The surface salinity largely depends on the flow condition of the Kuroshio Current and, occasionally, the discharge of the Changjiang River during the summer season. Many oysters and ark shell farms are located, which discharge a large amount of organic matter into the water column in the Tongyeong coastal area. The undegraded organic matter then accumulates in the local and nearby sediments. The east Geoje coastal area, in contrast, has far fewer farms and is only occasionally influenced by the discharge of the Nakdong River through the Nakdong Estuary Barrage that was built in 1987. The climate in the Tongyeong-Geoje coastal area is strongly influenced by seasonal events. Strong north to northwest winds and minimal precipitation are characteristics of winter (December to February), while the relatively weak south to southeast winds and heavy precipitation are typical of summer (June to September). The typhoon-induced heavy rainfall and mixing of the whole water column could be another feature of summer to autumn, especially August to September.

## Environmental parameters

Water samples for nutrient analysis were filtered with a 0.45-µm-pore-size, 25-mm-diameter GF/F filter (Whatman^®^, Clifton, NJ, USA) on board, placed in acid-cleaned polyethylene bottles, treated with HgCl_2_, and then stored at –20 °C for further analysis. Ammonia, nitrate plus nitrite (NOx), phosphate, and silicate concentrations were determined in the laboratory using a flow injection autoanalyzer (QuikChem 8000; Lachat Instruments, Loveland, CO, USA). The nutrient concentrations were calibrated using standard brine solutions (CSK Standard Solutions; Wako Pure Chemical Industries, Osaka, Japan). For chlorophyll-*a* (Chl*-a*) measurements, 0.5 L of seawater was immediately filtered through a 0.45-µm-pore-size, 25-mm-diameter GF/F filter (Whatman^®^, Clifton, NJ, USA) on board and stored at –20 °C until further analysis. Chl*-a* was measured using a Turner-designed fluorometer (10-AU; Turner BioSystems, Sunnyvale, CA, USA) following the extraction of the filtered material with 90% acetone for 24 h in the dark ^1^. Briefly, after the first measurement (R_b_), 1 N HCl was added in each sample. Then, the samples were measured again to gain R_a_. Chl-*a* concentration was calculated with the following equation :

Chl-*a* = F_d_ × [r / (r – 1)] × (R_b_ – R_a_) × (V_a_ / V_s_)

where:

F_d_ : calibration coefficient using Chl-*a* standard (Turner Designs)

R_b_ : sample fluorescence before acidification with HCl

R_a_ : sample fluorescence after acidification with HCl

r : maximum acid ratio (R_b_/R_a_) of pure Chl-*a* standard

V_a_ : extraction volume (mL)

V_s_ : filtered volume (L).

To identify and enumerate phytoplankton populations, 0.5-L water samples were collected in polyethylene bottles and fixed with 0.5% Lugol’s solution on board. These samples were then concentrated to approximately 50 mL by decanting the supernatant as described by Sournia ^2^. A Sedgewick-Rafter counting chamber was used to estimate the number of phytoplankton using a light microscope (Carl Zeiss, Germany).

### Supplementary Tables

#### Table S1. Pearson’s correlation coefficients between size fraction-specific bacterial diversity indices (alpha-diversity, species richness, and evenness) and abiotic environmental parameters. Alpha-diversity, species richness, and evenness were measured by Shannon's diversity index, the Chao1 richness estimator, and Simpson’s evenness index, respectively. *** *P* < 0.001; ** *P* < 0.01, * *P* < 0.05. |*r*| > 0.5 is highlighted in bold type.

|  | **Size fraction** | **NOx**  **[μM]** | **Phosphate**  **[μM]** | **Silicate**  **[μM]** | **Ammonia**  **[μM]** | **Temperature**  **[°C]** | **Salinity**  **[psu]** | **pH** | **DO**  **[%]** | **Chl-*a***  **[μg l^-1^]** |
| --- | --- | --- | --- | --- | --- | --- | --- | --- | --- | --- |
| **Alpha-diversity** | **FL** | **0.65^***^** | **0.65^***^** | **0.58^***^** | -0.17 | -0.25^*^ | 0.00 | **-0.68^***^** | **-0.78^***^** | -0.21 |
|  | **MP** | **0.71^***^** | **0.67^***^** | **0.57^***^** | -0.07 | -0.25^*^ | 0.15 | -0.46^***^ | **-0.67^***^** | -0.41^***^ |
|  | **NP** | **0.51^***^** | **0.52^***^** | 0.40^***^ | -0.13 | -0.42^***^ | 0.36^***^ | -0.47^***^ | **-0.53^***^** | -0.12 |
| **Richness** | **FL** | **0.65^***^** | **0.70^***^** | **0.62^***^** | -0.02 | -0.36^***^ | 0.20 | **-0.58^***^** | **-0.81^***^** | -0.26^*^ |
|  | **MP** | **0.65^***^** | **0.58^***^** | **0.55^***^** | 0.05 | -0.15 | 0.11 | -0.46^***^ | **-0.58^***^** | -0.26^*^ |
|  | **NP** | **0.67^***^** | **0.62^***^** | **0.50^***^** | -0.11 | -0.31^**^ | 0.16 | **-0.51^***^** | **-0.65^***^** | -0.29^**^ |
| **Evenness** | **FL** | 0.22^*^ | 0.13 | 0.15 | -0.14 | 0.22^*^ | -0.34^**^ | **-0.64^***^** | -0.35^**^ | -0.06 |
|  | **MP** | 0.39^***^ | 0.32^**^ | 0.27^*^ | -0.31^**^ | -0.25^*^ | 0.29^**^ | -0.34^**^ | -0.44^***^ | -0.21 |
|  | **NP** | -0.03 | 0.00 | -0.02 | 0.01 | -0.02 | 0.12 | -0.28^*^ | -0.18 | 0.04 |

Table S2. perMANOVA analysis based on Bray-Curtis dissimilarities using bacterial abundance data to evaluate the extend of size fraction, sampling depth, sampling station, and sampling month on bacterial community compositions. *P*-values were obtained using 999 permutations.

|  | **Degree of freedom** | **Pseudo *F*** | ***P*** |
| --- | --- | --- | --- |
| **Size fraction** | 2 | 40.90 | <0.001 |
| **Sampling depth** | 2 | 2.93 | <0.001 |
| **Sampling station** | 5 | 1.43 | <0.05 |
| **Sampling month** | 4 | 11.96 | <0.001 |

#### Table S3. Proportions of significant correlations (edges) among OTUs in different size fractions, environmental factors, and phytoplankton populations against the total number of possible correlations between node types.

| **Parameters** | **Avg. Nodes^*^** | **FL** | **NP** | **MP** | **Env** | **Phytoplankton** |
| --- | --- | --- | --- | --- | --- | --- |
| **FL** | 202±46 | 6.5±2.8% |  |  |  |  |
| **NP** | 133±51 | 1.2±1.7% | 1.2±1.1% |  |  |  |
| **MP** | 40±17 | 0.6±0.9% | 0.5±0.9% | 0.9±0.7% |  |  |
| **Env** | 9±1 | 8.1±3.4% | 3.1±2.8% | 1.8±1.8% | 19.6±7.2% |  |
| **Phytoplankton** | 7±4 | 0±0% | 0±0% | 0.2±0.4% | 1.2±2.4% | 0±0% |

Abbreviations: Avg. Nodes, averaged nodes; Env, environmental factors.

^*^Avg. Nodes: average nodes of FL, NP, and MP were the average values and their standard deviations of those OTUs with a relative abundance higher than 0.01% per sample and observed in more than 70% of samples in the same size fraction of each month. Average nodes of phytoplankton were the averaged values and their standard deviations of those phytoplankton genera observed in more than 70% of samples in each month.

#### Table S4. Average proportions of significant correlations (edges) between specific phyla and environmental parameters against the total number of possible correlations.

| **Parameters** | **Avg. Nodes^*^** | ***Actinobacteria*** | ***Bacteroidetes*** | ***Planctomycetes*** | ***Proteobacteria*** | **SAR406** | ***Verrucomicrobia*** | **Env** |
| --- | --- | --- | --- | --- | --- | --- | --- | --- |
| ***Actinobacteria*** | 11±1 | 13.0±7.1% |  |  |  |  |  |  |
| ***Bacteroidetes*** | 58±14 | 5.9±4.1% | 6.4±3.0% |  |  |  |  |  |
| ***Planctomycetes*** | 12±7 | 8.8±9.0% | 4.1±2.9% | 16.7±25.0% |  |  |  |  |
| ***Proteobacteria*** | 134±38 | 8.1±4.4% | 4.6±1.9% | 7.1±7.8% | 6.0±2.6% |  |  |  |
| **SAR406** | 9±4 | 18.2±14.8% | 5.5±3.2% | 17.8±27.1% | 10.9±7.2% | 43.8±35.8% |  |  |
| ***Verrucomicrobia*** | 17±4 | 8.8±6.8% | 4.0±2.4% | 9.1±8.7% | 5.3±3.1% | 10.9±9.0% | 8.7±5.1% |  |
| **Env** | 9±1 | 12.6±7.4% | 6.3±3.0% | 11.9±8.6% | 9.9±4.2% | 19.9±15.0% | 6.9±4.0% | 25.4±12.5% |

Abbreviations: Avg. Nodes, average nodes; Env, environmental factors.

^*^Avg. Nodes: average nodes of each phylum were the average values and their standard deviations of those observed OTUs that belonged to each phylum.

Table S5. The list of module hubs in each month.

| Sampling Month | OTUs ID | Module affiliation | Size fraction | Averaged relative abundance (monthly) | C score | Z score | Node degree | phylum | class | order | family | genus |
| --- | --- | --- | --- | --- | --- | --- | --- | --- | --- | --- | --- | --- |
| 7 | FL_Otu00012 | surface | FL | 1.82 | 0 | 2.2684 | 20 | Proteobacteria | Alphaproteobacteria | Rhodospirillales | Rhodospirillaceae | AEGEAN-169_marine_group |
| 7 | FL_Otu00030 | surface | FL | 3.61 | 0 | 1.7254 | 17 | Proteobacteria | Alphaproteobacteria | Rhodobacterales | Rhodobacteraceae | Amylibacter |
| 7 | FL_Otu00037 | surface | FL | 0.52 | 0 | 1.5445 | 16 | Proteobacteria | Gammaproteobacteria | Cellvibrionales | Halieaceae | OM60(NOR5)_clade |
| 7 | FL_Otu00091 | mid&bot | FL | 0.29 | 0.5461 | 1.5577 | 41 | Proteobacteria | Gammaproteobacteria | Salinisphaerales | Salinisphaeraceae | ZD0417_marine_group |
| 7 | FL_Otu00157 | surface | FL | 0.27 | 0 | 1.9064 | 18 | Bacteroidetes | Flavobacteriia | Flavobacteriales | Cryomorphaceae | Owenweeksia |
| 7 | FL_Otu00393 | mid&bot | FL | 0.08 | 0.5071 | 1.5577 | 55 | Verrucomicrobia | Arctic97B-4_marine_group | Arctic97B-4 marine_group | Arctic97B-4_marine_group | unclassified |
| 8 | FL_Otu00001 | mid&bot | FL | 24.95 | 0.0512 | 1.9535 | 38 | Proteobacteria | Alphaproteobacteria | SAR11_clade | SAR11 subclade I | Candidatus_Pelagibacter |
| 8 | FL_Otu00019 | surface | FL | 1.48 | 0.2637 | 1.6751 | 32 | Proteobacteria | Alphaproteobacteria | Rickettsiales | SAR116_clade | Candidatus_Puniceispirillum |
| 8 | FL_Otu00026 | mid&bot | FL | 1.02 | 0.0928 | 2.1393 | 41 | Proteobacteria | Alphaproteobacteria | Rickettsiales | SAR116_clade | unclassified |
| 8 | FL_Otu00028 | mid&bot | FL | 2.24 | 0.1078 | 1.5819 | 35 | Bacteroidetes | Flavobacteriia | Flavobacteriales | Flavobacteriaceae | NS4_marine_group |
| 8 | FL_Otu00088 | mid&bot | FL | 0.57 | 0 | 2.0464 | 38 | Bacteroidetes | Flavobacteriia | Flavobacteriales | Flavobacteriaceae | NS5_marine_group |
| 8 | FL_Otu00180 | mid&bot | FL | 0.2 | 0 | 1.5819 | 33 | Actinobacteria | Actinobacteria | Micrococcales | Microbacteriaceae | Candidatus_Aquiluna |
| 8 | NP_Otu00191 | mid&bot | NP | 0.6 | 0 | 2.3251 | 41 | Planctomycetes | OM190 | OM190 | OM190 | unclassified |
| 9 | FL_Otu00005 | all | FL | 2.19 | 0 | 1.7771 | 30 | Proteobacteria | Gammaproteobacteria | Oceanospirillales | ZD0405 | unclassified |
| 9 | FL_Otu00010 | all | FL | 1.73 | 0 | 1.7771 | 30 | Proteobacteria | Gammaproteobacteria | Oceanospirillales | SAR86_clade | unclassified |
| 9 | FL_Otu00025 | all | FL | 1.12 | 0.0408 | 1.7508 | 48 | SAR406_clade | SAR406_clade | SAR406_clade | SAR406_clade | unclassified |
| 9 | FL_Otu00072 | all | FL | 0.11 | 0 | 2.9128 | 42 | Proteobacteria | Betaproteobacteria | Methylophilales | Methylophilaceae | OM43_clade |
| 9 | FL_Otu00094 | all | FL | 0.14 | 0 | 1.8718 | 31 | Proteobacteria | Alphaproteobacteria | Rhodobacterales | Rhodobacteraceae | unclassified |
| 9 | FL_Otu00112 | all | FL | 0.16 | 0 | 1.5879 | 28 | Gemmatimonadetes | Gemmatimonadetes | BD2-11 terrestrial_group | unclassified | unclassified |
| 9 | FL_Otu00152 | all | FL | 0.49 | 0.1172 | 1.6154 | 48 | Chloroflexi | SAR202_clade | SAR202_clade | SAR202_clade | unclassified |
| 9 | FL_Otu00154 | all | FL | 0.26 | 0.4996 | 1.9927 | 35 | Proteobacteria | Betaproteobacteria | Burkholderiales | Alcaligenaceae | MWH-UniP1_aquatic_group |
| 9 | FL_Otu00199 | all | FL | 0.18 | 0 | 1.6154 | 45 | Proteobacteria | Gammaproteobacteria | Oceanospirillales | SAR86_clade | unclassified |
| 9 | FL_Otu00200 | all | FL | 0.07 | 0 | 1.9664 | 32 | Proteobacteria | SPOTSOCT00m83 | SPOTSOCT00m83 | SPOTSOCT00m83 | unclassified |
| 9 | FL_Otu00221 | all | FL | 0.12 | 0 | 1.6154 | 45 | Proteobacteria | Alphaproteobacteria | Rickettsiales | SAR116_clade | unclassified |
| 9 | FL_Otu00287 | all | FL | 0.08 | 0.2293 | 1.6831 | 53 | Proteobacteria | Alphaproteobacteria | Rickettsiales | SAR116_clade | unclassified |
| 9 | FL_Otu00589 | all | FL | 0.03 | 0.282 | 1.5476 | 53 | Proteobacteria | Deltaproteobacteria | Desulfuromonadales | GR-WP33-58 | unclassified |
| 9 | FL_Otu00772 | all | FL | 0.04 | 0 | 1.6825 | 29 | Proteobacteria | Gammaproteobacteria | unclassified | unclassified | unclassified |
| 9 | NP_Otu00194 | all | NP | 0.29 | 0.4778 | 1.6247 | 38 | Proteobacteria | Deltaproteobacteria | Bdellovibrionales | Bdellovibrionaceae | OM27_clade |
| 10 | FL_Otu00007 | all | FL | 5.34 | 0.2076 | 1.6152 | 17 | Proteobacteria | Alphaproteobacteria | Rhodobacterales | Rhodobacteraceae | unclassified |
| 10 | FL_Otu00009 | all | FL | 2.61 | 0 | 1.8279 | 16 | Proteobacteria | Alphaproteobacteria | Rhodobacterales | Rhodobacteraceae | Ascidiaceihabitans |
| 10 | FL_Otu00034 | all | FL | 0.38 | 0 | 1.8593 | 18 | Bacteroidetes | Flavobacteriia | Flavobacteriales | Flavobacteriaceae | NS5_marine_group |
| 10 | FL_Otu00037 | all | FL | 1.18 | 0 | 1.6152 | 15 | Proteobacteria | Gammaproteobacteria | Cellvibrionales | Halieaceae | OM60(NOR5)_clade |
| 10 | FL_Otu00091 | mid&bot | FL | 0.4 | 0 | 1.911 | 28 | Proteobacteria | Gammaproteobacteria | Salinisphaerales | Salinisphaeraceae | ZD0417_marine_group |
| 10 | FL_Otu00123 | mid&bot | FL | 0.21 | 0 | 1.5457 | 25 | Proteobacteria | Alphaproteobacteria | Rhodospirillales | Rhodospirillaceae | AEGEAN-169_marine_group |
| 10 | FL_Otu00140 | all | FL | 0.43 | 0.1975 | 1.5228 | 18 | Bacteroidetes | Flavobacteriia | Flavobacteriales | Flavobacteriaceae | unclassified |
| 10 | FL_Otu00150 | all | FL | 0.05 | 0.2378 | 3.0371 | 29 | Proteobacteria | Gammaproteobacteria | KI89A_clade | unclassified | unclassified |
| 10 | FL_Otu00242 | all | FL | 0.06 | 0.1723 | 2.0276 | 21 | Proteobacteria | Betaproteobacteria | unclassified | unclassified | unclassified |
| 10 | FL_Otu00383 | mid&bot | FL | 0.06 | 0 | 1.6675 | 26 | SAR406_clade | SAR406_clade | SAR406_clade | SAR406_clade | unclassified |
| 10 | NP_Otu00007 | all | NP | 3.67 | 0.1327 | 2.3566 | 14 | Proteobacteria | Alphaproteobacteria | Rhodobacterales | Rhodobacteraceae | unclassified |
| 10 | NP_Otu00031 | surface | NP | 0.63 | 0 | 4.1582 | 11 | Bacteroidetes | Flavobacteriia | Flavobacteriales | NS9_marine_group | unclassified |
| 10 | NP_Otu00032 | mid&bot | NP | 0.73 | 0 | 1.8995 | 13 | Proteobacteria | Gammaproteobacteria | Salinisphaerales | Salinisphaeraceae | ZD0417_marine_group |
| 10 | NP_Otu00052 | surface | NP | 0.95 | 0 | 1.5829 | 6 | Bacteroidetes | Flavobacteriia | Flavobacteriales | NS9_marine_group | unclassified |
| 10 | NP_Otu00058 | all | NP | 0.57 | 0.1528 | 1.7512 | 12 | Proteobacteria | Gammaproteobacteria | Oceanospirillales | OM182_clade | unclassified |
| 10 | NP_Otu00069 | mid&bot | NP | 0.46 | 0 | 1.6564 | 12 | Proteobacteria | Deltaproteobacteria | Desulfobacterales | Nitrospinaceae | Nitrospina |
| 10 | NP_Otu00091 | mid&bot | NP | 0.39 | 0 | 1.6564 | 12 | Proteobacteria | Gammaproteobacteria | Salinisphaerales | Salinisphaeraceae | ZD0417_marine_group |
| 10 | NP_Otu00095 | mid&bot | NP | 0.12 | 0 | 1.6564 | 12 | Proteobacteria | Gammaproteobacteria | E01-9C-26 marine_group | unclassified | unclassified |
| 12 | FL_Otu00023 | all | FL | 1.74 | 0 | 1.7451 | 37 | Actinobacteria | Acidimicrobiia | Acidimicrobiales | OM1_clade | Candidatus_Actinomarina |
| 12 | FL_Otu00034 | all | FL | 0.82 | 0 | 1.7451 | 37 | Bacteroidetes | Flavobacteriia | Flavobacteriales | Flavobacteriaceae | NS5_marine_group |
| 12 | FL_Otu00056 | all | FL | 0.46 | 0 | 1.6592 | 36 | Bacteroidetes | Flavobacteriia | Flavobacteriales | NS9_marine_group | unclassified |
| 12 | FL_Otu00057 | all | FL | 0.26 | 0 | 1.831 | 38 | Bacteroidetes | Flavobacteriia | Flavobacteriales | Flavobacteriaceae | NS5_marine_group |
| 12 | FL_Otu00183 | all | FL | 0.3 | 0 | 1.5733 | 35 | Bacteroidetes | Flavobacteriia | Flavobacteriales | Cryomorphaceae | Owenweeksia |
| 12 | FL_Otu00228 | mid&bot | FL | 0.26 | 0 | 1.6136 | 37 | SAR406_clade | SAR406_clade | SAR406_clade | SAR406_clade | unclassified |

### Supplementary Figures

#### Fig. S1. Six sampling sites in the Tongyeong-Geoje coastal area of South Korea. For each station, the original sampling ID is designated. Latitude, longitude, and water depth of each sampling station are specified in the table.

####
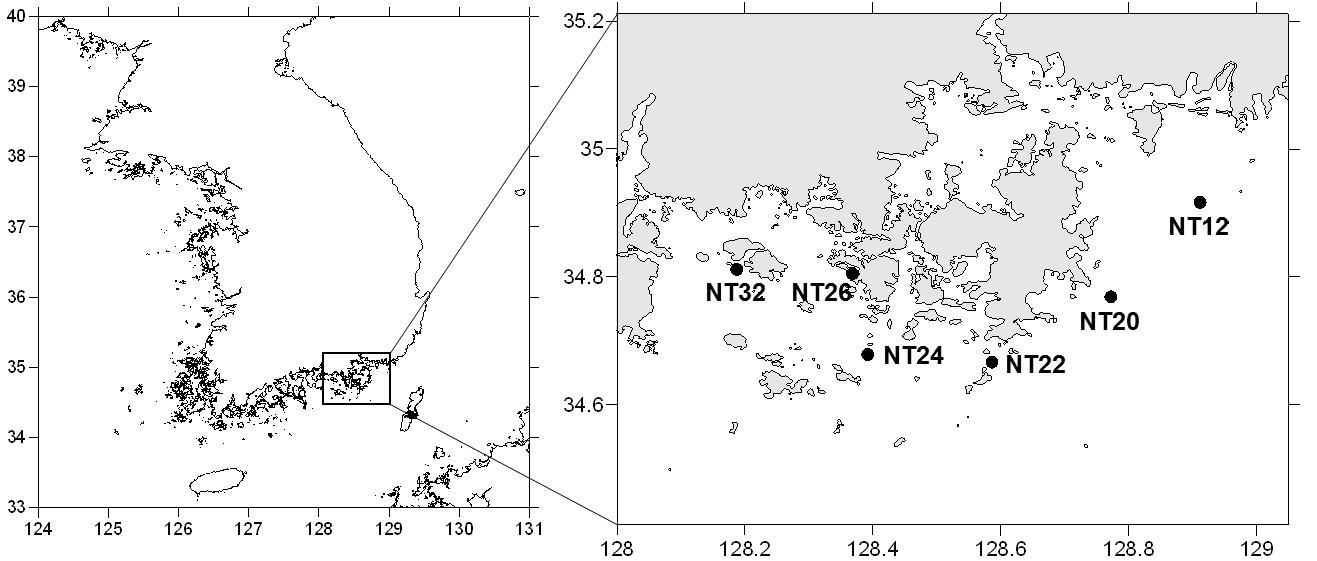


| **Station** | **Latitude (N)** | **Longitude (E)** | **Depth [m]** |
| --- | --- | --- | --- |
| NT12 | 34.917 | 128.911 | 50 |
| NT20 | 34.769 | 128.772 | 53 |
| NT22 | 34.667 | 128.586 | 65 |
| NT24 | 34.679 | 128.392 | 56 |
| NT26 | 34.805 | 128.368 | 30 |
| NT32 | 34.812 | 128.187 | 48 |

#### Fig. S2. Changes in environmental parameters in the seawater over the half-year of 2016 in the Southern Coast of Korea: a, temperature; b, salinity; c, NOx; d, phosphate; e, silicate; f, ammonia; g, Chl*-a*; h, pH; i, DO (dissolved oxygen).


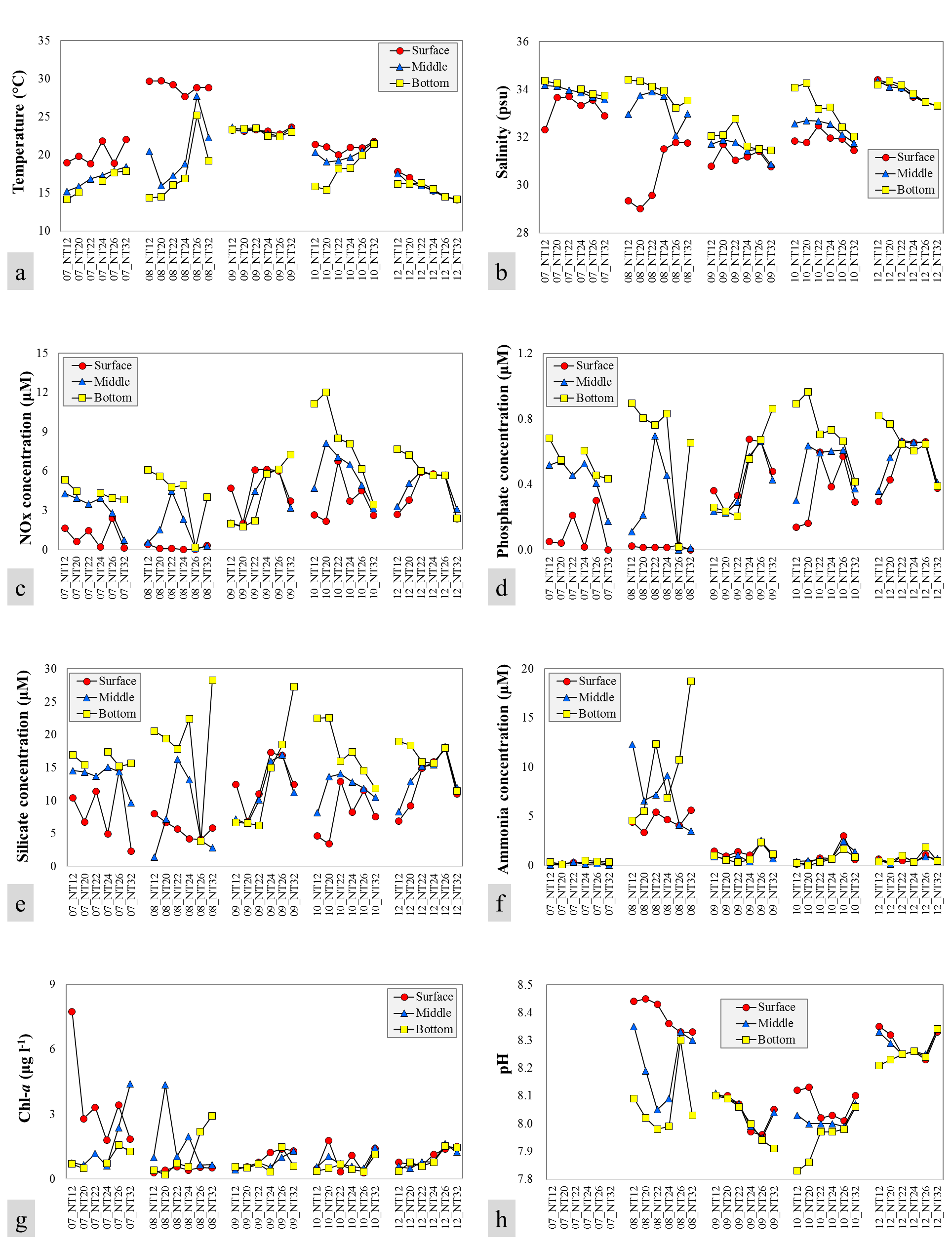


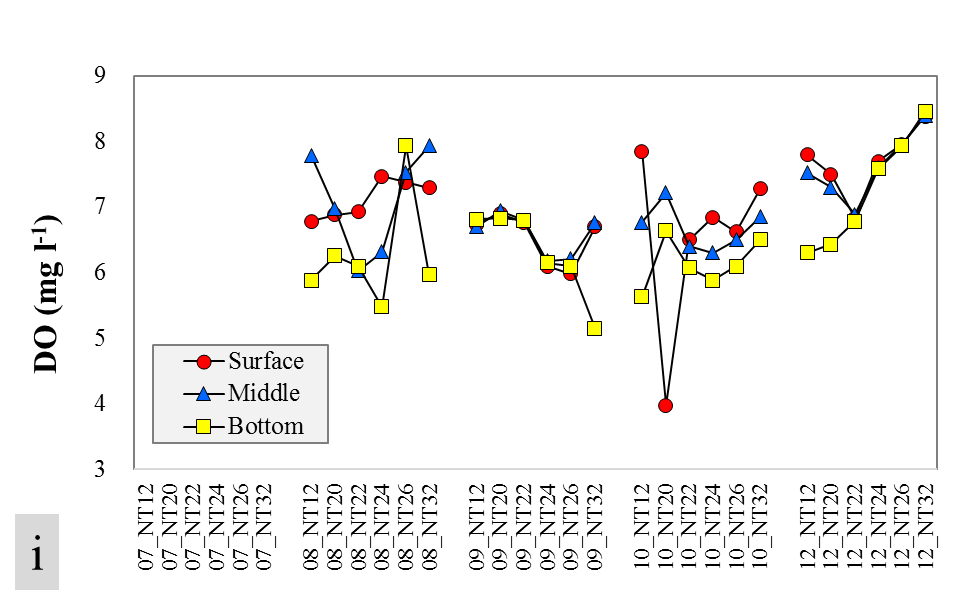


#### Fig. S3. Phytoplankton abundance observed with a microscope. The six most abundant phytoplankton genera in a) surface, b) middle, and c) bottom water layers are listed.


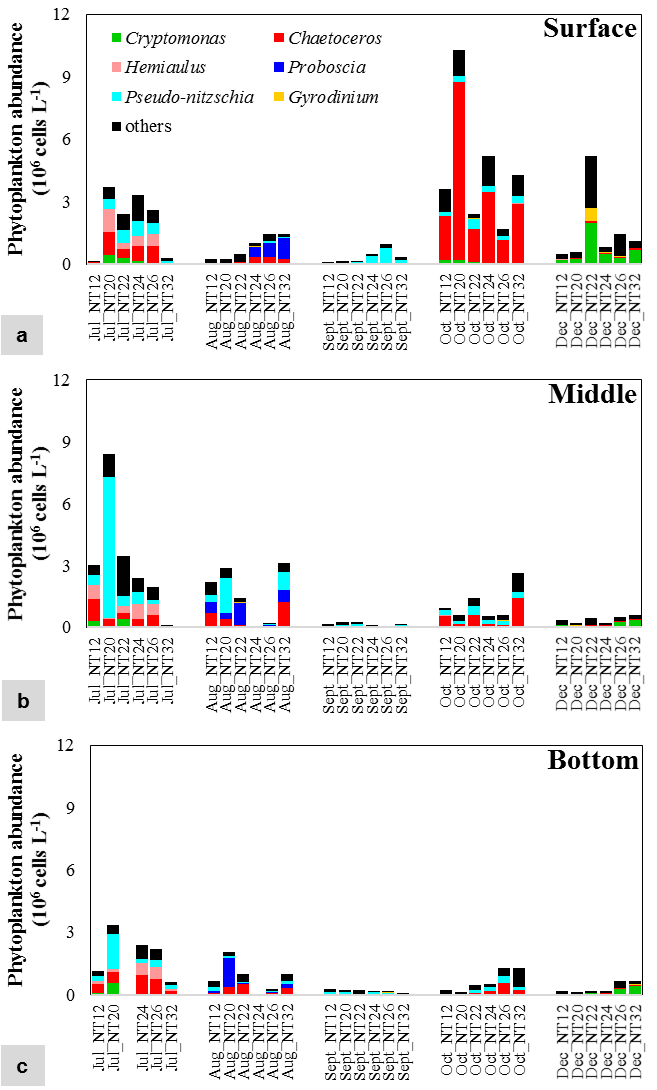


#### Fig. S4. Bacterial community diversity (a-d), species richness (e-h), and evenness (i-l). Panels a, e, and i were constructed with samples from all size fractions (total samples). The remaining other panels were constructed with samples from each size fraction to compare three indices according to water depths (from the surface to the bottom).


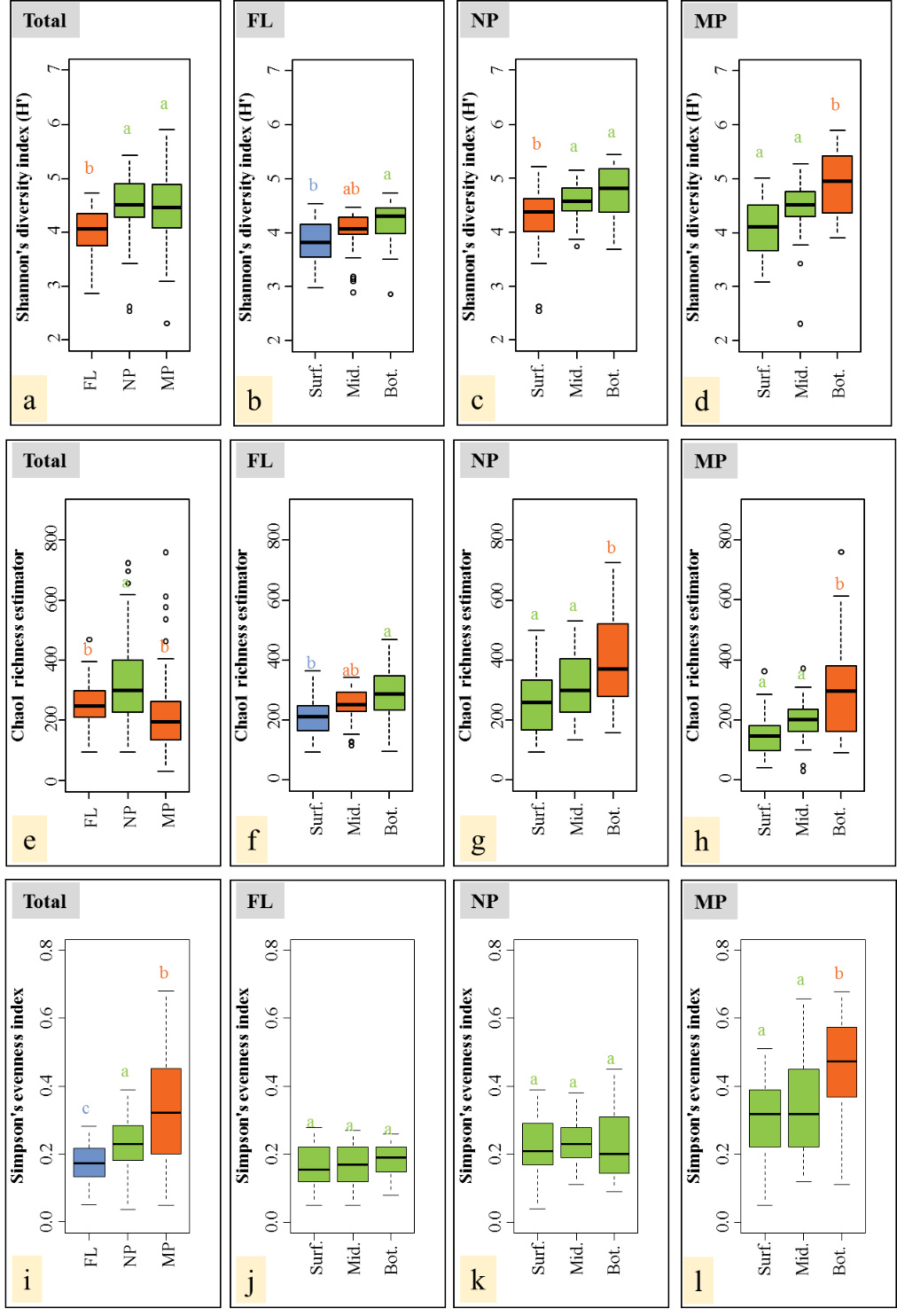


#### Fig. S5. Non-metric multidimensional scaling (NMDS) representing the distance between samples by size fraction. The NMDS was constructed with a Bray–Curtis distance matrix. Gray open circles: OTUs. The colored symbols indicate the samples from different size fractions each month. The goodness of fit of the NMDS was measured using a 2-dimensional stress of 0.19.


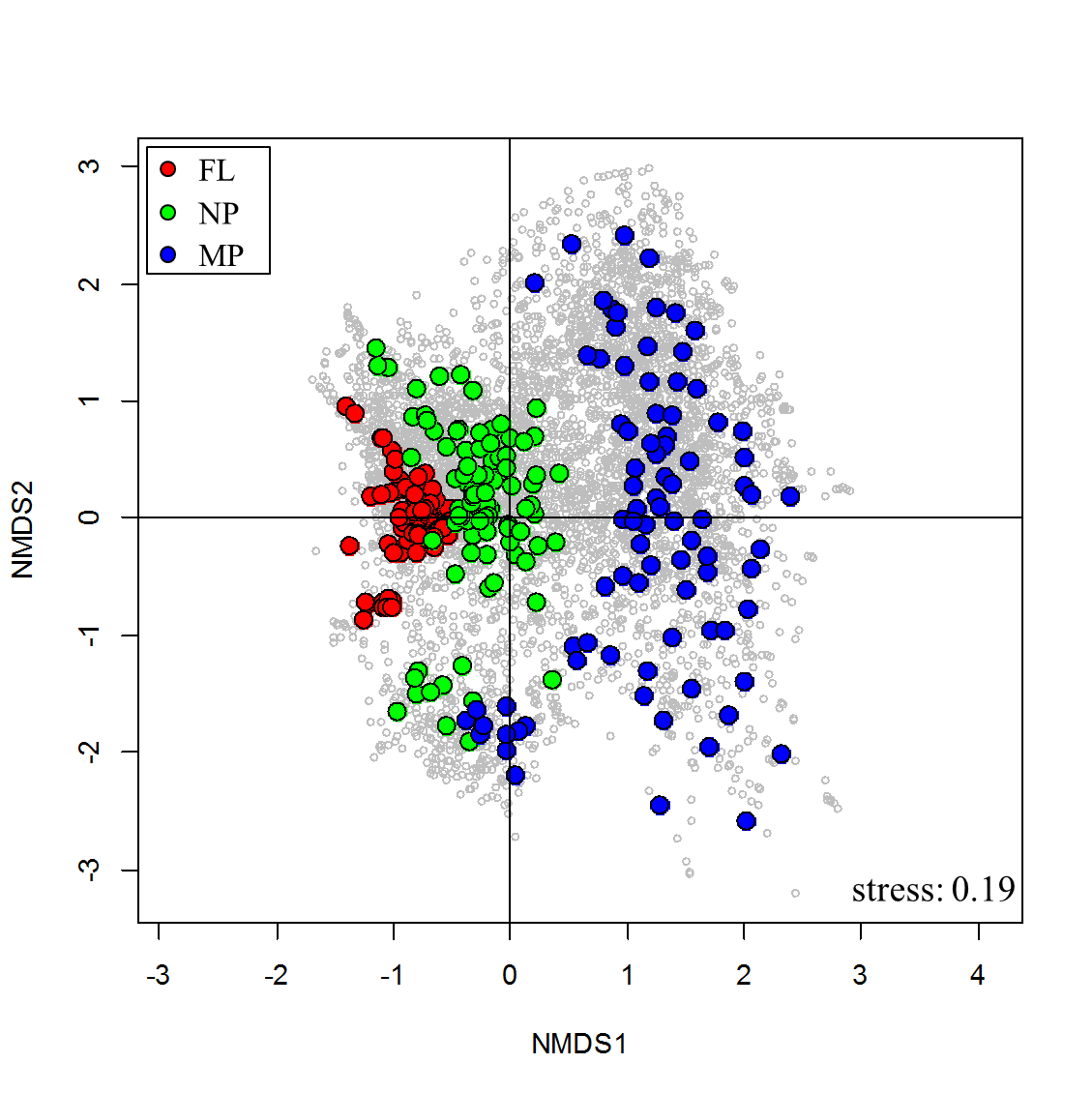


#### Fig. S6. Non-metric multi-dimensional scaling (NMDS) ordinations of the bacterial 16S rRNA gene community in FL, NP, and MP samples. a) FL fraction; b) NP fraction; and c) MP fraction. Gray open circles indicate individual bacterial OTUs. The goodness of fit of the NMDS was measured using a 2-dimensional stress of 0.14, 0.16, and 0.18 in FL, NP, and MP fractions, respectively.


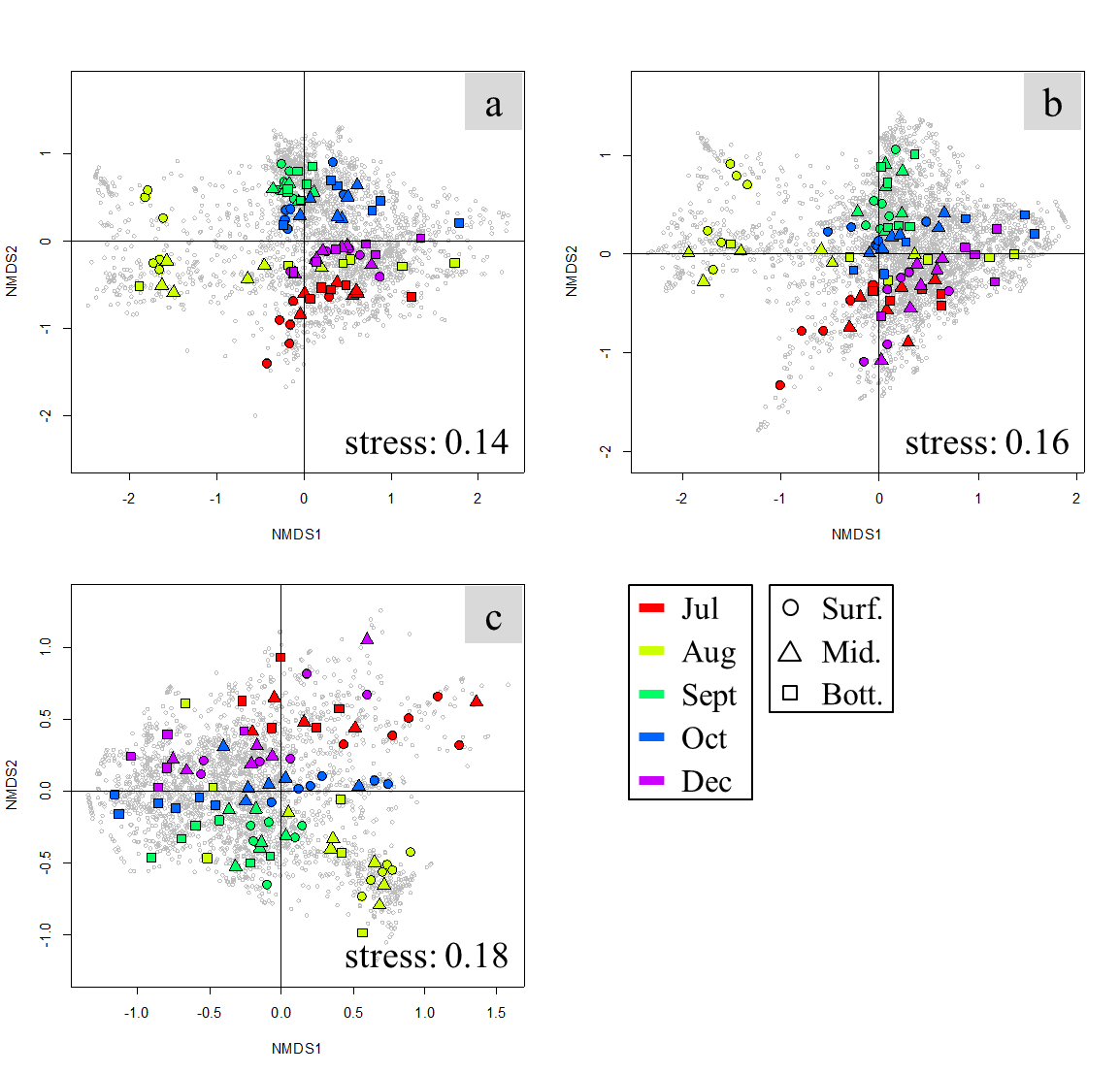


#### Fig. S7. The spatiotemporal changes of bacterial community compositions across the sampling period. OTUs were grouped at the level of class. Only those of classes that contributed more than 1% to total bacteria are listed. The monthly collected FL, NP, and MP samples are listed from left to right.


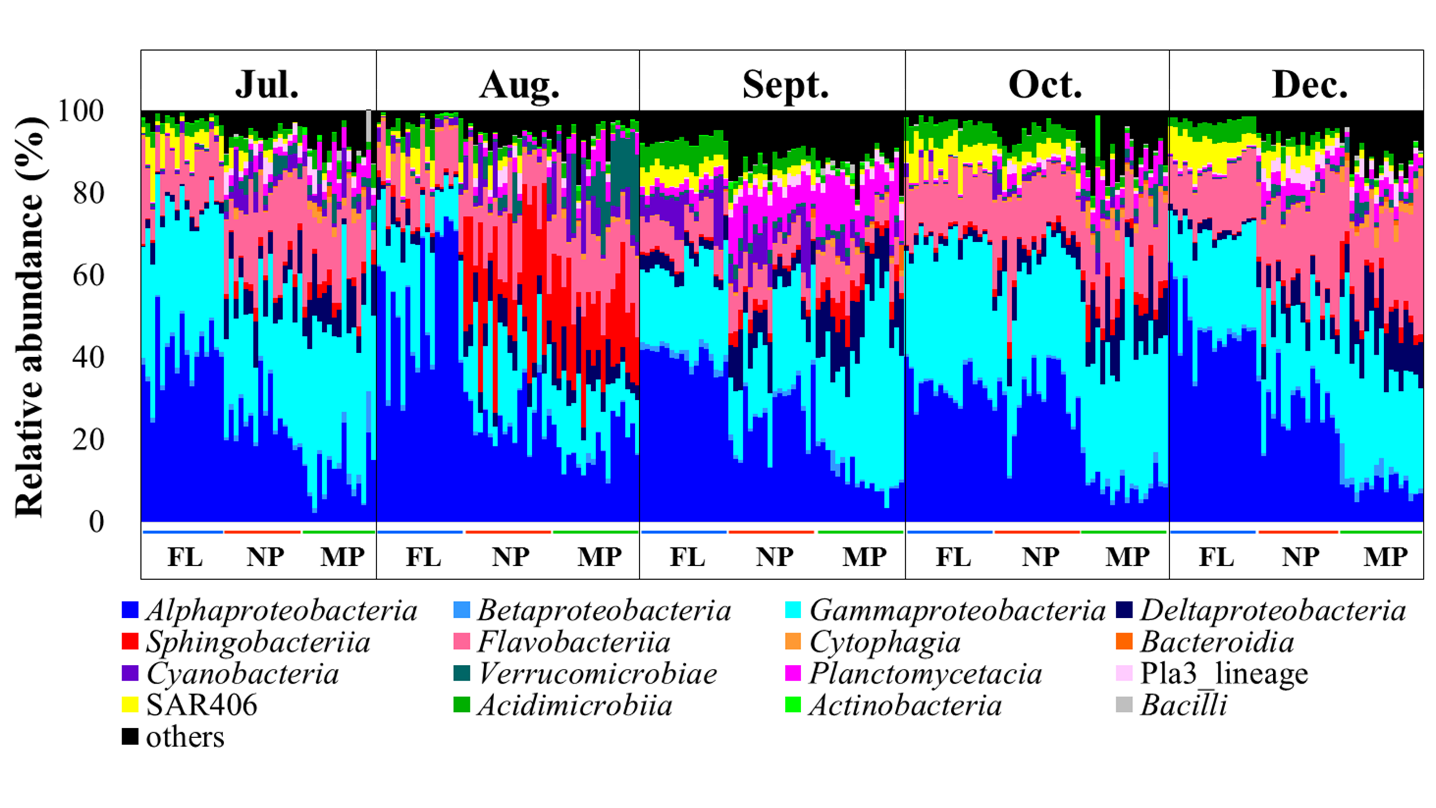


#### Fig. S8. Comparison of the FAPROTAX-generated functional profile of FL/NP/MP bacteria in different water depths and months.


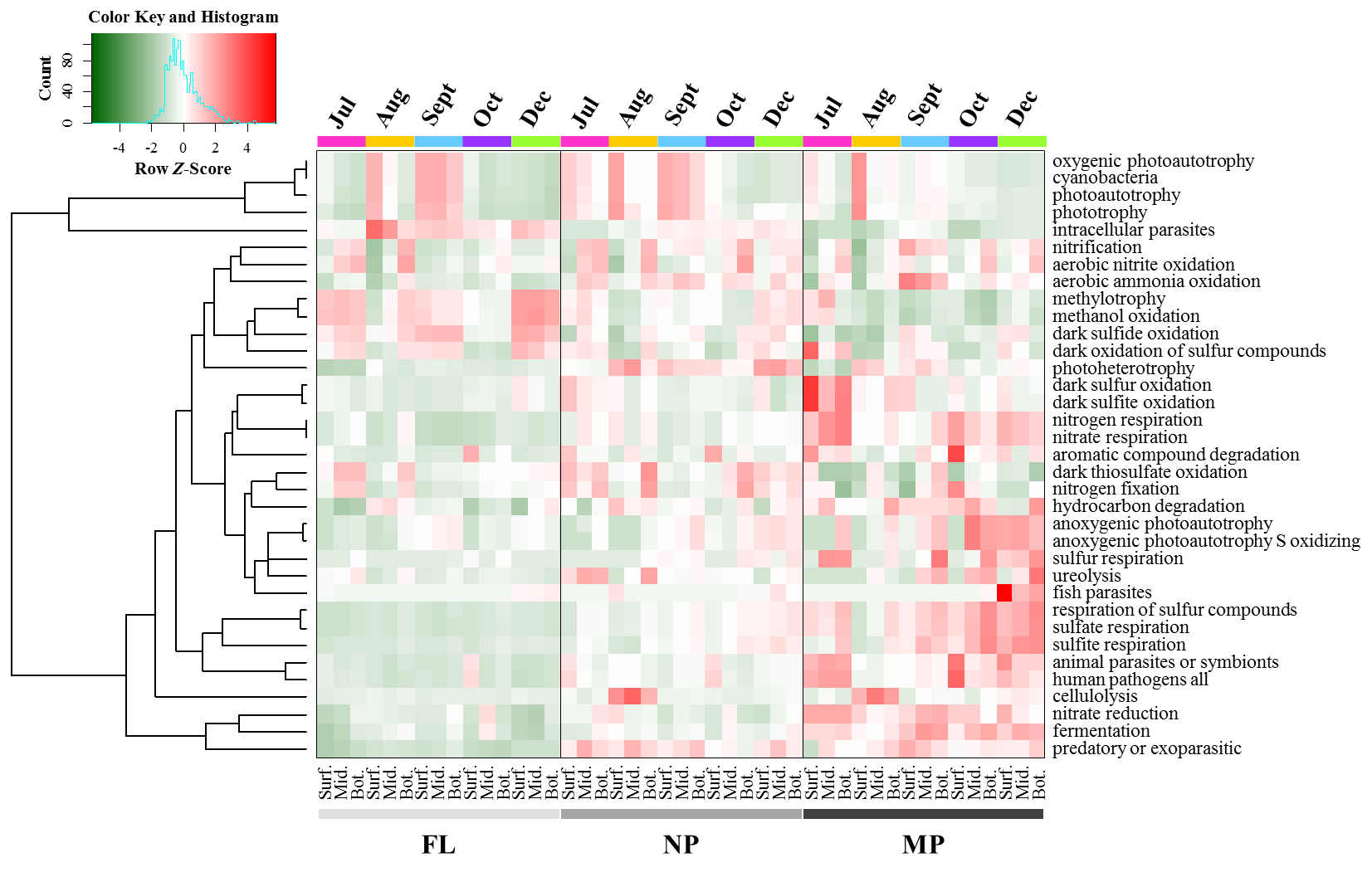


### Supplementary References

1. Parsons, T. R., Y. Maita & C. M. Lalli. A Manual of Chemical and Biological Methods for Seawater Analysis. Pergamon Press, Oxford, p. 173. (1984)
2. Sournia, A. Phytoplankton Manual. UNESCO, Paris, p. 337. (1978)
